# Size-Dependent Axonal Bouton Dynamics following Visual Deprivation *In Vivo*

**DOI:** 10.1016/j.celrep.2017.12.065

**Published:** 2018-01-29

**Authors:** Rosanna P. Sammons, Claudia Clopath, Samuel J. Barnes

**Affiliations:** 1 Department of Neuroscience, Physiology and Pharmacology, University College London, 21 University St., London WC1E 6DE, UK; 2 Department of Biomedical Engineering, Imperial College London, South Kensington Campus, London SW7 2AZ, UK; 3 Division of Brain Sciences, Department of Medicine, Imperial College London, Hammersmith Hospital Campus, Du Cane Road, London W12 0NN, UK

**Keywords:** visual cortex, axonal bouton, population coupling, presynaptic, plasticity, GCaMP, network, homeostasis, sensory deprivation, LTP

## Abstract

Persistent synapses are thought to underpin the storage of sensory experience, yet little is known about their structural plasticity *in vivo*. We investigated how persistent presynaptic structures respond to the loss of primary sensory input. Using *in vivo* two-photon (2P) imaging, we measured fluctuations in the size of excitatory axonal boutons in L2/3 of adult mouse visual cortex after monocular enucleation. The average size of boutons did not change after deprivation, but the range of bouton sizes was reduced. Large boutons decreased, and small boutons increased. Reduced bouton variance was accompanied by a reduced range of correlated calcium-mediated neural activity in L2/3 of awake animals. Network simulations predicted that size-dependent plasticity may promote conditions of greater bidirectional plasticity. These predictions were supported by electrophysiological measures of short- and long-term plasticity. We propose size-dependent dynamics facilitate cortical reorganization by maximizing the potential for bidirectional plasticity.

## Highlights

• The range of persistent axonal bouton sizes is reduced following visual deprivation
• Bouton sizes move toward the mean in a size-dependent manner
• Bouton plasticity is accompanied by a reduced range of correlated network activity
• Deprived cortex exhibits greater bidirectional functional presynaptic plasticity

## Graphical Abstract

**Figure:**
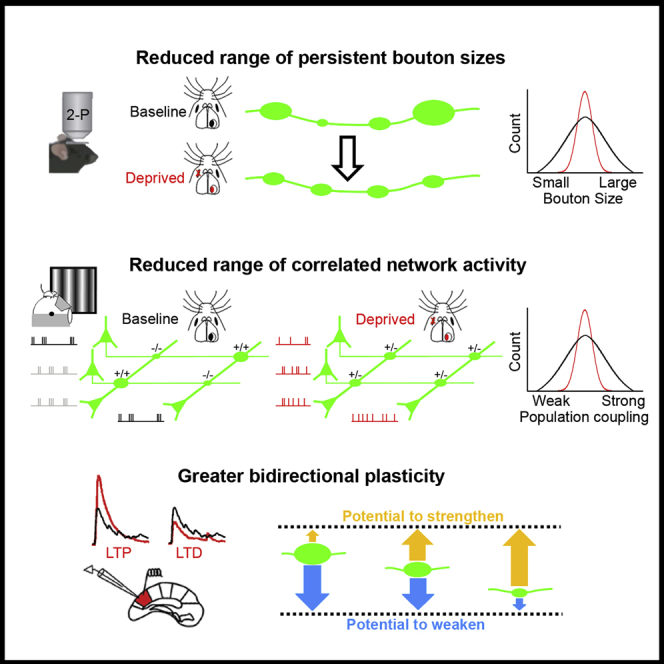

## Teaser

Sammons et al. investigate the structural dynamics of persistent axonal boutons. Although central to many theories of sensory processing, persistent synapses remain understudied. Visual deprivation collapses the distribution of bouton sizes toward the mean. Bouton plasticity is accompanied by a reduced range of correlated network activity and greater bidirectional plasticity.

## Introduction

Sensory experience modifies synaptic strength and connectivity between cortical neurons (Cheetham et al., 2007, Albieri et al., 2015). Theoretical studies suggest that the distribution of synaptic strengths both processes sensory input and provides a substrate to encode experience (Barbour et al., 2007, Stepanyants and Escobar, 2011). If sensory experience is stored in the synaptic strength distribution, then what happens to this distribution when primary sensory input is removed?

Diminished sensory experience can induce synapse loss but its effect on surviving synapses is less clear (Barnes et al., 2015a, Barnes et al., 2017). Conditions that drive synapse loss may weaken surviving synapses (Albieri et al., 2015, Barnes et al., 2015a) or trigger homeostatic changes in strength (Barnes et al., 2017). Alternately, prolonged loss of sensory activity may shape the distribution of surviving synaptic strengths. In this scenario, theoretical studies predict the loss of patterned sensory input would narrow the range of synaptic strengths/weights (Barbour et al., 2007). To test between these scenarios, we removed the contralateral visual drive to mouse monocular primary visual cortex (V1m), and we studied the dynamics of persistent presynaptic structures.

Persistent presynaptic structures are understudied. This is surprising given their abundance in the cortex (Qiao et al., 2016). More attention has been paid to transient synaptic structures, which are thought to be critical for circuit reorganization (De Paola et al., 2006, Keck et al., 2008, Barnes and Cheetham, 2014). Persistent synaptic structures exhibit fluctuations in size both *in vivo* (Grillo et al., 2013, Keck et al., 2013, Barnes et al., 2017) and in reduced preparations (Minerbi et al., 2009, Meyer et al., 2014). We used fluctuations in the size of persistent axonal boutons as a proxy for synaptic strength *in vivo* over time (Canty and De Paola, 2011, Grillo et al., 2013). This approach is supported by work showing a strong correlation between the size of a dendritic spine or axonal bouton and its synaptic strength (Murthy et al., 2001, Noguchi et al., 2011, Cheetham et al., 2014).

Here we use *in vivo* two-photon (2P) imaging to measure fluctuations in the size of persistent axonal boutons in L2/3 of adult V1m following monocular enucleation. We find a reduction in the range of bouton sizes in deprived cortex. After deprivation, large boutons shrink and small boutons grow so that the distribution of bouton sizes narrows. These size-dependent dynamics are accompanied by a reduction in the range of correlated network activity in L2/3 of awake animals. Computational modeling, based on our *in vivo* data, suggests that size-dependent dynamics may be driven by a reduced range of correlated network activity. Furthermore, our model suggests that bouton dynamics increase the potential for bidirectional plasticity (i.e., greater potential for both synaptic strengthening and weakening). Electrophysiological experiments support the predictions of our simulation, and they show greater short- and long-term presynaptic plasticity after deprivation. Our results suggest that size-dependent bouton dynamics may generate conditions that benefit reorganization after deprivation by enhancing the flexibility of synapses to undergo both synaptic strengthening and weakening.

## Results

### Size-Dependent Bouton Dynamics

**Figure 1:**
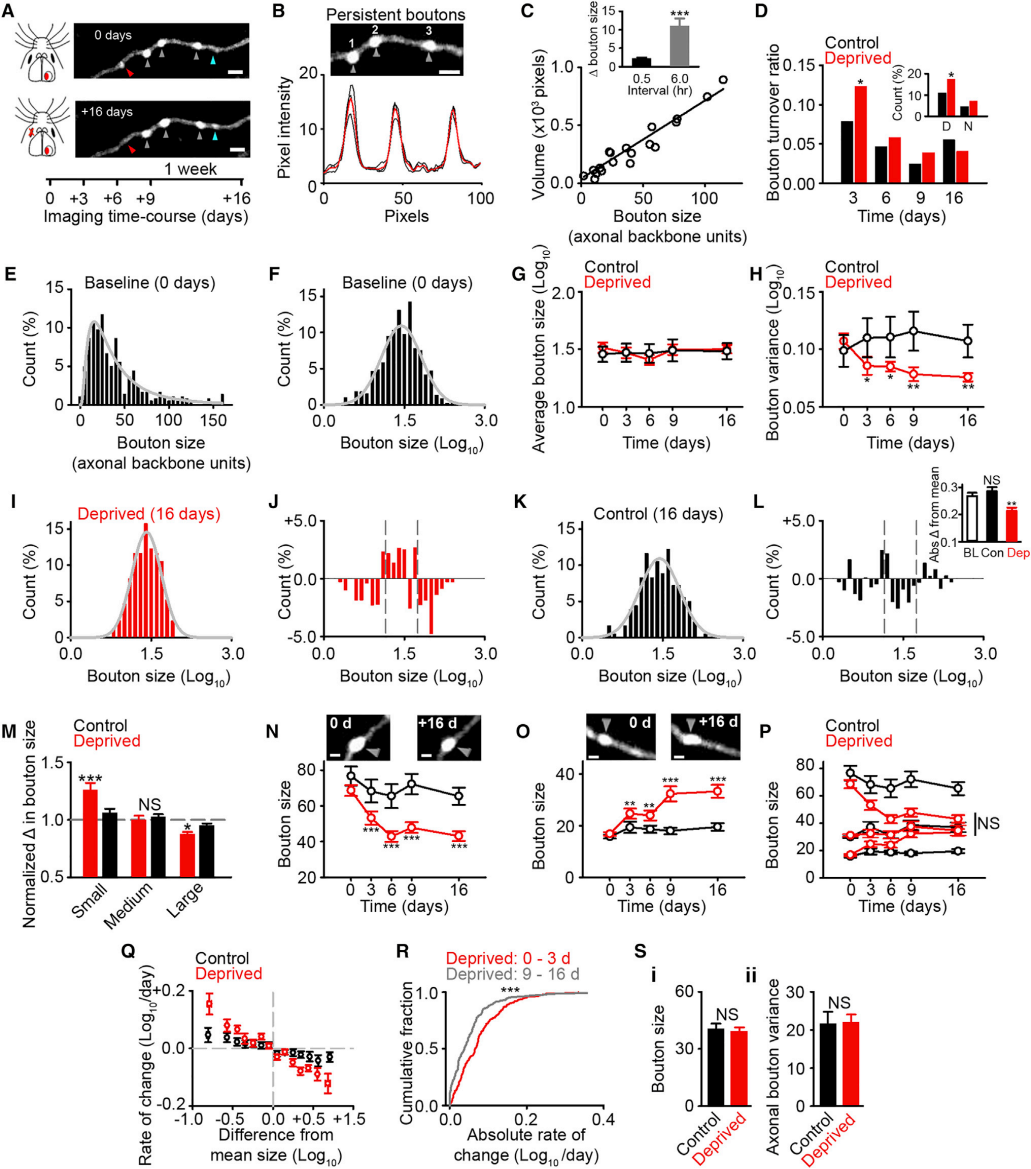
Size-Dependent Bouton Dynamics in Deprived Cortex (A) Time course of 2P imaging paradigm and enucleation. Axon from L2/3 in V1m of *Thy1*-GFP mouse is shown. Arrows indicate lost (red), new (cyan), and persistent boutons (gray). Scale bar, 2 μm. (B) Intensity profiles from three persistent boutons above. Red, average taken at 3 depths. Scale bar, 2 μm. (C) Comparison of analysis program versus summed volume. (Inset) Bouton size over short (0.5 hr) versus longer periods (6 hr) is shown. (D) Turnover ratios for deprived and control cortex. (Inset) Percentage of disappearing (D) and new (N) boutons at 3 days is shown. (E) Histogram of bouton size in axonal backbone units (0 day). (F) Histogram of Log_10_ bouton sizes from (E). (G) Average persistent bouton size per region in control and deprived cortices. (H) Variance of persistent axonal boutons per region in control and deprived cortices. (I and K) Log_10_ bouton sizes in deprived (I, 16 days) and control (K, 16 days) cortices. (J and L) Difference in deprived (J) and control (L) Log_10_ bouton sizes between 16 days and 0 day. Gray lines, 25^th^ and 75^th^ percentiles. (L, inset) Absolute difference in bouton size from Log_10_ mean at baseline (0 day, black, open), in control cortex (16 days, black, filled) and deprived cortex (16 days, red, filled), is shown. (M) Normalized change in size of small, medium, and large boutons relative to baseline in control and deprived cortices. (N–P) Time course of size-dependent bouton dynamics in deprived (red) and control (black) cortices for large (N), small (O), and all boutons (P). Scale bar, 1 μm. (Q) Rate of change per day between imaging sessions for boutons at different sizes, relative to the mean size, in control and deprived cortices. (R) Absolute rate of change per day for boutons in deprived cortex between 0 and 3 days or 9 and 16 days. (S) Persistent bouton size (i) and the variance (ii) of bouton sizes in axonal backbone units in control and deprived cortices. For statistical comparisons and n values, see Table S1. NS, not significant; ^∗^p < 0.05, ^∗∗^p < 0.01, and ^∗∗∗^p < 0.001. Error bars, mean and SEM.

We repeatedly imaged axonal boutons through a cranial window implanted over V1m in adult *Thy1*-GFP mice (Feng et al., 2000) before and after monocular enucleation (Figure 1A). Using published criteria, we defined axonal swellings as boutons (Grillo et al., 2013), which were considered persistent if present at each imaging time point (Figure 1A). Fluctuations in persistent bouton size were measured using custom software and quantified as normalized axonal backbone units. Bouton measurements correlated with manual measurements of summed bouton volume (Figures 1B and 1C), and, although stable within imaging sessions, they fluctuated over longer periods (Figure 1C, inset). Measurements of bouton turnover found a small increase 3 days after deprivation (Figure 1D), attributable to more disappearing boutons (Figure 1D, inset). However, after day 3, turnover rates were similar to control values (Figure 1D).

Dendritic spines can undergo homeostatic increases in size following sensory deprivation (Keck et al., 2013, Barnes et al., 2017); whether axonal boutons do too is unclear. We measured the size of persistent boutons following deprivation as a proxy for synaptic strength (Murthy et al., 2001, Cheetham et al., 2014, Grillo et al., 2013). Similar to work investigating dendritic spine sizes *in vivo* (Loewenstein et al., 2011), we found the bouton size distribution during baseline was positively skewed, reminiscent of a log-normal distribution (Figure 1E). A histogram of Log_10_ bouton sizes demonstrated that the bouton size distribution was approximated by a log-normal function (Figure 1F). We compared the average bouton size per imaged region at each time point to baseline, and we found no net shift in average bouton size following deprivation (Figure 1G). Instead, the variance of bouton sizes (per imaged region) was reduced; this was not the case in control cortex (Figure 1H).

A reduction in the size of large boutons without any change in smaller boutons would lead to a reduced range of bouton sizes, as would an increase in the size of smaller boutons with no change in large boutons. Such changes may have important implications, as synapse size influences the potential for synaptic plasticity (Matsuzaki et al., 2004). Thus, we next asked how the bouton size distribution changed after deprivation. We subtracted the deprived distribution (16 days) (Figure 1I) from the baseline distribution (Figure 1F), and we observed fewer large and small boutons in the distribution tails and more boutons around the median (Figures 1I and 1J). This was not the case for time-matched controls (Figures 1K and 1L). To quantify the reduction in bouton variance, we took the absolute difference of each bouton from the mean of the Log_10_ population (Figure 1L, inset). The absolute difference of each bouton was lower in deprived cortex than baseline (Figure 1L, inset). This was not the case for time-matched controls (Figure 1L, inset). We next asked how individual bouton changes narrow the distribution of bouton sizes. We binned boutons by their size before deprivation (see the Supplemental Experimental Procedures), and we examined the change in size over time. We saw a strong inverse correlation between initial bouton size and change in size in deprived cortex (Figure 1M). In contrast, large and small boutons in controls showed similar changes to medium-sized boutons (Figure 1M). The changes in deprived cortex made boutons of different sizes become similar to the mean bouton size (Figures 1N–1P). Large boutons became smaller (Figures 1N and 1P) while small boutons increased in size (Figures 1O and 1P). This was not the case in control cortex for boutons binned identically (Figures 1N–1P).

We next investigated the temporal dynamics of boutons between imaging sessions. We focused on bouton size (measured with respect to the mean on each session) and the change in size expressed in the next session. We found a strong inverse relationship between bouton size and the rate of change in deprived cortex (Figure 1Q). Boutons with the greatest difference from the mean showed the most movement toward the mean on the next session. A weaker correlation was also evident in control cortex (Figure 1Q). Thus, subtle ongoing size-dependent structural dynamics occur in control cortex but are enhanced following deprivation. In deprived cortex, the rate of change slowed in later sessions, as boutons differed less from the mean (Figure 1R). Our results could not be explained by different structural properties prior to deprivation, as axons in deprived cortex had similar bouton size (Figure 1Si) and variance (Figure 1Sii) values to controls. Together our results show the range of bouton sizes is reduced in deprived cortex because boutons regress to the mean at rates proportional to their size. This reduces the difference between boutons that were previously either large (putative strong synapses) or small (putative weak synapses).

### Reduced Range of Correlated Network Activity

**Figure 2:**
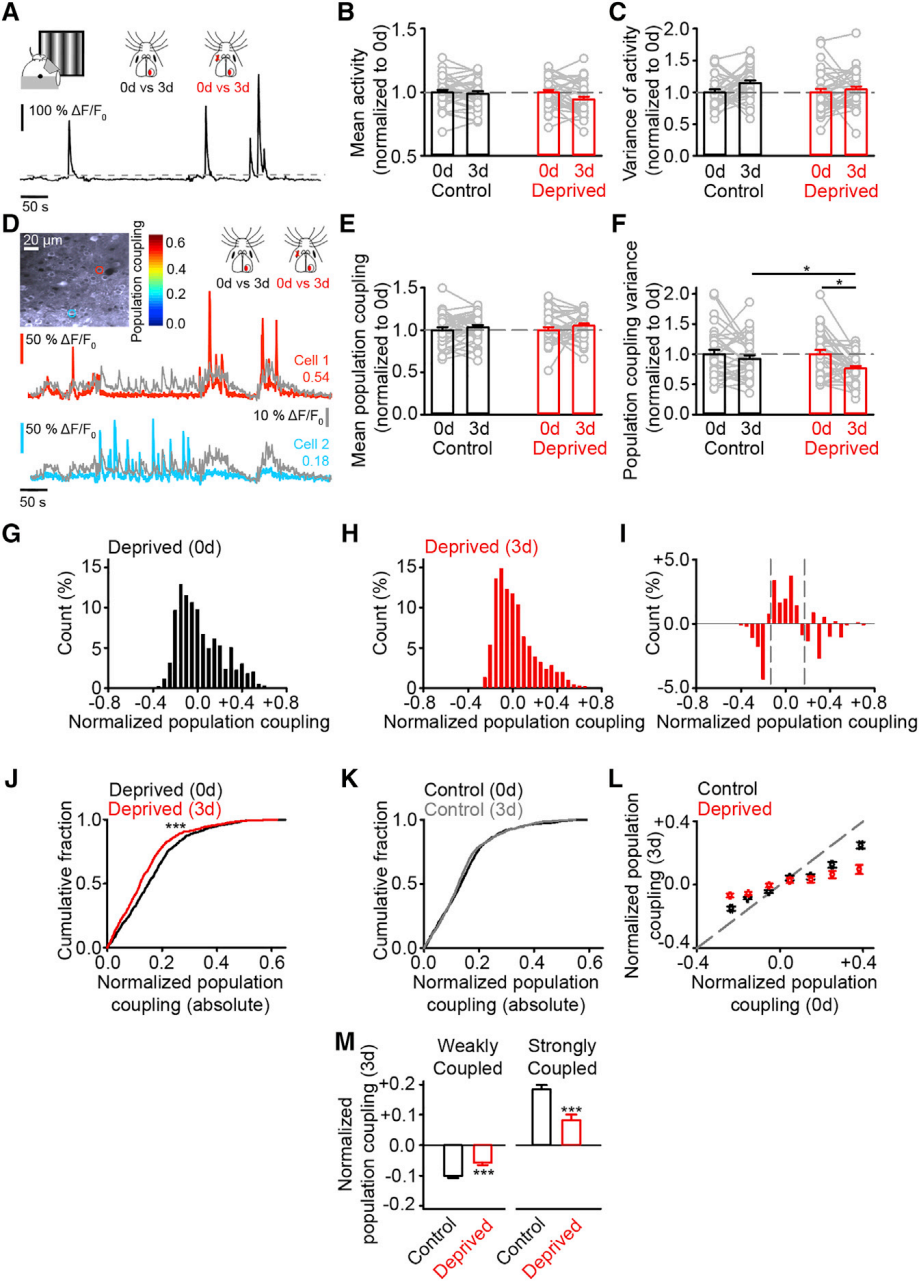
Reduced Range of Correlated Network Activity (A) Awake behaving imaging of GCaMP5 at 0 day or 3 days with example calcium trace. Gray dashed line is activity threshold. Scale bars, 100%ΔF/F_0_ and 50 s. (B and C) Mean (B) and variance (C) of activity in imaged region normalized to the baseline (0 day) for control and deprived cortices. Gray points show each region. (D) Example neurons highlighted by regions of interest (ROIs) with color corresponding to population coupling scale bar. GCaMP5 traces show population rate (gray trace), and color-coded traces are according to coupling scores (scores next to traces). Scale bars, individual cells: 50%ΔF/F_0_ and 50 s; average population rate trace: 10%ΔF/F_0_ and 50 s; region: 20 μm. (E and F) Mean (E) and variance (F) of population coupling scores per region normalized to the baseline (0 day) for control and deprived cortices. Gray points show each imaged region. (G and H) Normalized population coupling scores from deprived mice at 0 day (G) and 3 days (H). (I) Difference between deprived 3 days (from H) and deprived 0 day (from G) normalized population coupling distributions. Gray dashed lines are 25^th^ and 75^th^ percentiles. (J and K) Absolute normalized population coupling distributions in deprived (J) and control (K) cortices at 0 day and 3 days. (L) Normalized population coupling scores at 0 day and 3 days for neurons in control and deprived cortices. (M) Normalized population coupling scores for neurons in control and deprived cortices at 3 days that were either (left) weakly coupled or (right) strongly coupled to population activity when measured at 0 day. For statistical comparisons and n values, see Table S2. ^∗^p < 0.05 and ^∗∗∗^p < 0.001. Error bars, mean and SEM.

To estimate network conditions in deprived cortex, we used chronic 2P calcium imaging of GCaMP5 (Akerboom et al., 2012) in L2/3 of V1m in awake mice (Figure 2A). L2/3 excitatory neurons are a putative target and one source (see the Discussion: Measuring Persistent Boutons) of our imaged boutons (Feng et al., 2000), and thus they are a reasonable approximation of network activity for our boutons. Structural imaging data found size-dependent bouton plasticity at 3 days; thus, we compared network activity at day 0 to 3 days. We compared activity between deprived and control mice and within the same mice before and after deprivation (Figure 2A). We measured activity in each region using the integral of the calcium signal for each cell and normalizing this to the duration of imaging (Figure 2A). For both control and deprived animals, we normalized activity levels at all time points to baseline activity. We found the mean and the variance of activity in deprived cortex was similar both to baseline and time-matched control animals (Figures 2B and 2C). These results are consistent with homeostatic recovery of cortical activity following visual deprivation *in vivo* (Barnes et al., 2015b).

To determine how deprivation influences the correlation structure of cortical activity, we used the Pearson coupling statistic to generate population coupling scores for each neuron (Okun et al., 2015). We took the pairwise correlation value between each activity trace and the calcium trace of mean population activity (see the Supplemental Experimental Procedures) (Figure 2D). We normalized to the mean of the baseline, and we examined the mean and variance of population coupling scores in imaged regions over time. Changes in the mean population coupling score in an imaged region (relative to baseline) reflect the strength of correlations between neurons and mean population rate. In contrast, changes in the variance of population coupling scores reflect the range of correlated activity between different neurons and the mean population rate. Mean population coupling scores were similar to both baseline and time-matched controls (Figure 2E). In contrast, the variance of population coupling values in deprived cortex was reduced relative to baseline and control cortex (Figure 2F).

We combined all imaged regions by taking the difference between each neuron’s population coupling score in an imaged region and the average population coupling score of that region (see the Supplemental Experimental Procedures). Normalizing in this way allowed us to compare the distributions of population coupling values before (Figure 2G) and after deprivation (Figure 2H). We found fewer normalized population coupling scores in the tails of our distribution and more scores around the median after deprivation (Figure 2I). To quantify these changes, we repeated our normalization approach but instead took the absolute value of the normalized population coupling scores. This approach found the absolute difference from the population coupling mean for each neuron was reduced in deprived (Figure 2J), but not control, cortex (Figure 2K). Examining the behavior of individual neurons found cells with low normalized population coupling scores prior to deprivation had increased scores, relative to baseline and control cortex after deprivation (Figures 2L and 2M). Conversely, neurons with stronger population scores prior to deprivation weakened (Figures 2L and 2M). Thus, mean activity levels and mean population coupling scores were similar in deprived and control cortex, but the range of population coupling scores was reduced after deprivation. This occurred via a reduction in the strength of neurons with strong coupling and an increase in the strength of neurons with weak coupling.

### Network Simulation of Strength-Dependent Dynamics

**Figure 3:**
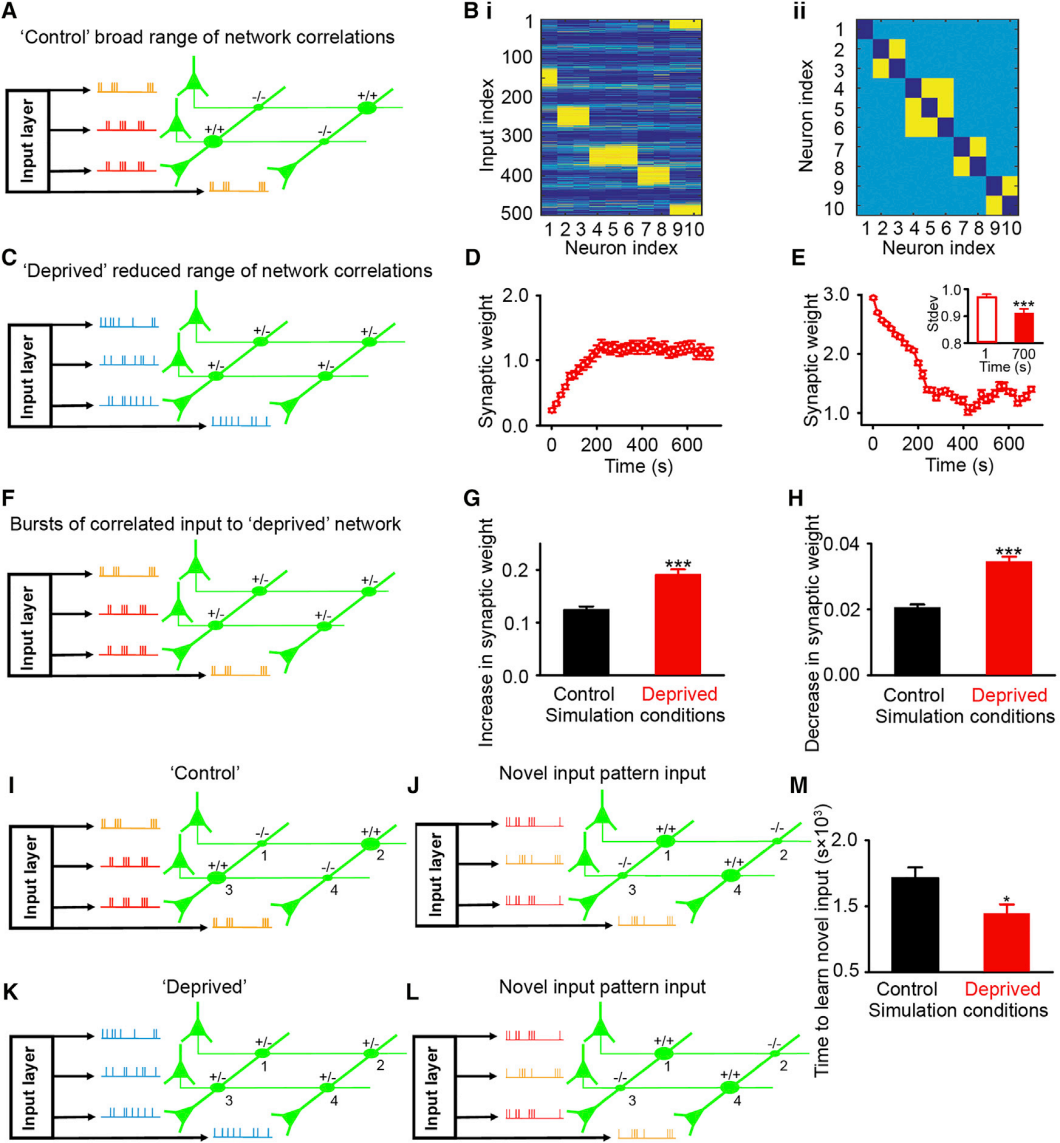
Network Simulation of Strength-Dependent Dynamics (A) Control simulation. Addition and subtraction symbols show weight strengths between neurons. (B) (i) Feedforward input weights into the neurons in the recurrent control network. Neurons 2 and 3 receive similar inputs, as do neurons 4–6, neurons 7 and 8, and neurons 9 and 10. Strong weights are yellow and weak weights are blue. (ii) Connection strengths from the presynaptic (x axis) to the postsynaptic neurons (y axis) within the control network. Neurons with correlated inputs are (yellow) bidirectionally connected, other neurons are (light blue) weakly connected, but not (dark blue) self-connected. (C) Deprived simulation with reduced range of correlated input. (D and E) The strength of weak weights (D) increases and the strength of strong weights (E) decreases (average of every 20^th^ time point). (Inset) SD of all weights measured at time points 1 and 700 is shown. (F) Schematic of plasticity simulation. Deprived model is presented with bursts of correlated input and the change in weights measured. (G and H) Deprived networks show greater plasticity when given bursts of positively (G) or negatively (H) correlated input. (I and J) Control network where weights (1–4) are established with highly correlated input (I) and then tested using novel input (J, i.e., a different set of neurons receives correlated inputs); weights 1 and 4 strengthen and weights 2 and 3 weaken. (K and L) Schematic of deprived network (K) and testing with novel input patterns (L). In the cartoon, synaptic weights 1 and 4 strengthen and synaptic weights 2 and 3 weaken after new input. (M) Deprived model learns novel input faster than control. For statistical comparisons and n values, see Table S3. ^∗^p < 0.05 and ^∗∗∗^p < 0.001. Error bars, mean and SEM.

To gain mechanistic insight into size-dependent dynamics, we used a network simulation based on activity measured *in vivo* and spike-timing-dependent plasticity rules (Clopath et al., 2010, Ko et al., 2013). Our simulation had a presynaptic input layer that sent feedforward excitation to postsynaptic neurons (Figures 3A and 3B; see the Supplemental Experimental Procedures). The model’s synaptic weights were trained with correlated input. Neurons with positively correlated input had strong connections, and those with negatively correlated input had weak connections (Figure 3Bii). We used the simulated weights to approximate pre-deprivation conditions. Then, in line with measurements of activity, we reduced the range of correlated input (Figure 3C), and we measured changes in weak (Figure 3D) and strong (Figure 3E) weights. Our model found strength-dependent weight dynamics (Figures 3D and 3E) mirrored structural changes *in vivo* (Figures 1N–1P). Reducing the range of correlated activity caused weak weights to strengthen (Figure 3D) and strong weights to weaken (Figure 3E) and, thus, reduced the range of weights (Figure 3E, inset). This occurred because strong weights are maintained by highly positive correlations. Thus, shifting to less correlated activity caused weakening. Similarly, weak weights were driven by negative correlations, so that less negatively correlated activity caused strengthening.

Both our *in vivo* imaging data and our network simulation found size/strength-dependent bouton dynamics resulted in a reduced range of synaptic weights/bouton sizes (Figures 1H, 3D, and 3E, inset). To determine the consequences for plasticity, we measured the change in weights after bursts of positively or negatively correlated input (see the Supplemental Experimental Procedures) (Figure 3F). Input bursts induced the most weight changes in our deprived model (Figures 3G and 3H). We reasoned that this increased potential for bidirectional plasticity may facilitate reorganization. Therefore, we compared the time taken for control (Figure 3I) and deprived (Figure 3K) networks to learn new input patterns (Figures 3I–3L). Simulations found deprived networks reached a saturated state of learning in response to novel input patterns faster than control networks (Figure 3M). Thus, our simulation suggests that reducing the range of correlated network activity may account for the reduced range of bouton sizes *in vivo* (likely leading to changes in both L2/3 input and output) and promote conditions of heightened bidirectional plasticity.

### Enhanced Bidirectional Plasticity

**Figure 4:**
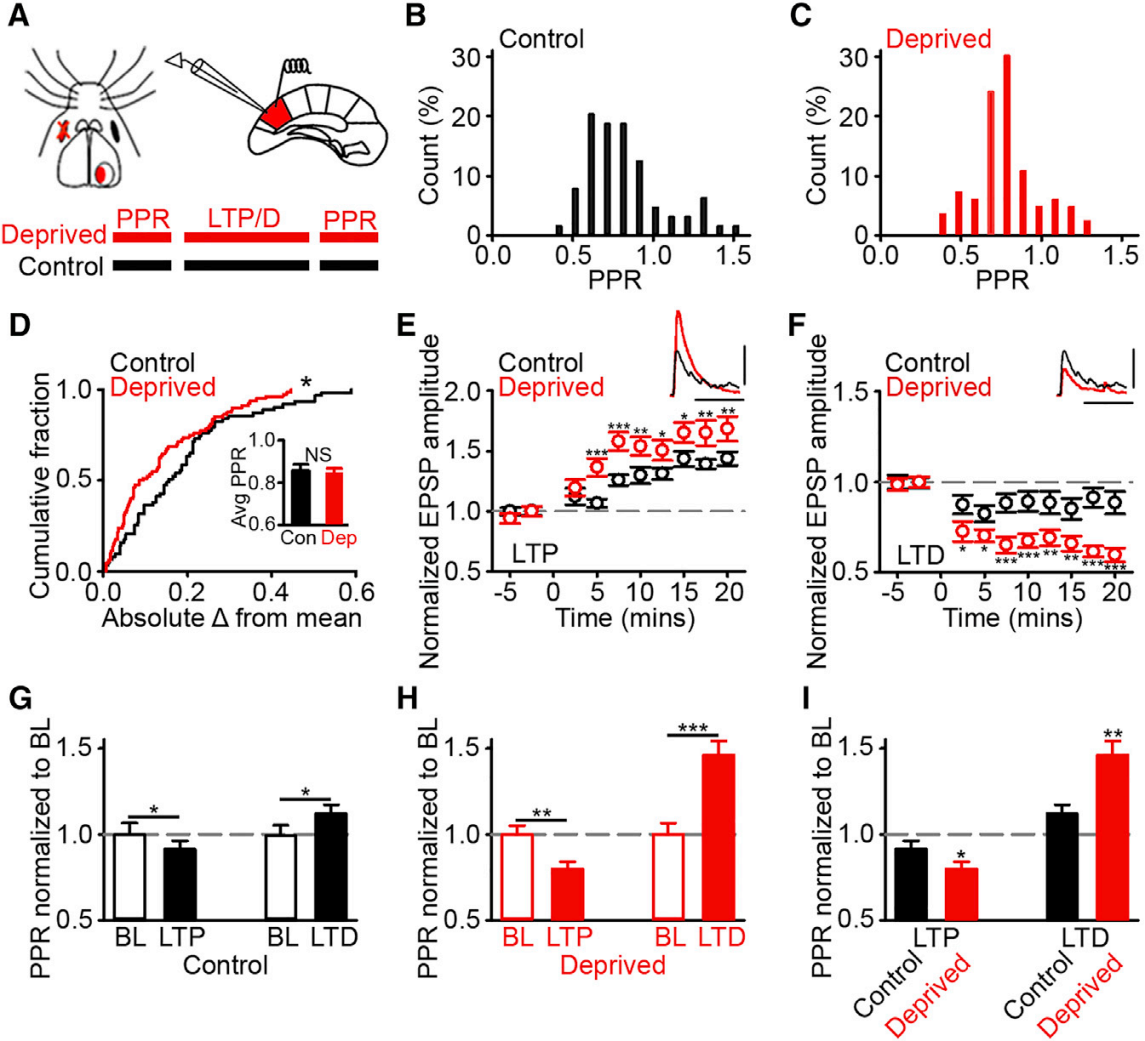
Enhanced Bidirectional Plasticity in Deprived Cortex (A) Schematic of deprived mouse and slice electrophysiology in V1m L2/3. Timeline for the characterization of PPR, LTP/LTD induction, and PPR re-testing is shown. (B and C) PPR in control (B) and deprived (C) cortices. (D) Absolute difference of PPRs from population mean and (inset) average PPR in control and deprived cortices. (E and F) LTP (E) and LTD (F) in deprived and control cortices. (Insets) Examples of excitatory postsynaptic potentials (EPSPs) from deprived cortex during baseline (E and F, black) and following either (red) LTP (E) or LTD (F) induction. Scale bars, 25 ms, 5 mV. (G and H) Normalized change in PPR relative to baseline in control (G) and deprived (H) cortices after (left) LTP or (right) LTD. (I) Change in PPR after induction of (left) LTP or (right) LTD in deprived and control cortex. Values are normalized to baseline. For statistical comparisons and n values, see Table S4. NS, not significant; ^∗^p < 0.05, ^∗∗^p < 0.01, and ^∗∗∗^p < 0.001. Error bars, mean and SEM.

Our network simulation predicted greater potential for bidirectional plasticity after size-dependent dynamics. The potential for synapses in deprived cortex to undergo enhanced synaptic strengthening and weakening may benefit reorganization. We used electrophysiology to examine these predictions. We made acute slices from deprived visual cortex at a time when imaging found the greatest reduction in bouton variance (16 days), and then we made whole-cell recordings from L2/3 pyramidal neurons (Figure 4A). To test the potential for neurons in L2/3 to undergo presynaptic plasticity, we first measured the paired pulse ratio (PPR) as an estimate of presynaptic release probability (Pr) (Regehr, 2012). For each neuron, we characterized the average PPR at a range of stimulation frequencies (1–10 Hz), inducing both paired pulse facilitation (PPF) and depression (PPD) (Figures 4B and 4C). The average PPR value was similar in control and deprived cortices (Figures 4B–4D, inset). However, the range of PPRs was reduced after deprivation so that the absolute difference of each PPR from the mean was less in deprived than control cortex (Figure 4D).

We next examined the degree of presynaptic plasticity expressed in deprived cortex by inducing a presynaptic form of either long-term potentiation (LTP) or depression (LTD), known to modify the PPR in cortical tissue (see the Supplemental Experimental Procedures) (Boudkkazi et al., 2007). Both LTP and LTD were greater in deprived than control cortex (Figures 4E and 4F). The induction of presynaptic LTP/LTD modified the PPR in control cortex, and these changes occurred in line with predicted shifts in Pr (Boudkkazi et al., 2007) (Figure 4G). After LTP induction, we observed a modest increase in PPD consistent with an increase in Pr, while LTD induction led to a small increase in PPF consistent with a decrease in Pr (Figure 4G). The changes in PPR after either LTP or LTD induction in deprived cortex were greater than those in control cortex (Figures 4H and 4I). Thus, our electrophysiology experiments support our network simulation suggesting size-dependent bouton dynamics are accompanied by greater bidirectional plasticity in deprived cortex.

## Discussion

We find persistent axonal boutons undergo size-dependent dynamics that reduce the range of bouton sizes in deprived cortex. This process is accompanied by a reduced range of correlated network activity that when modeled predicts greater potential for bidirectional plasticity (i.e., synaptic strengthening and synaptic weakening). Electrophysiological experiments support these predictions, suggesting that size-dependent bouton dynamics may promote conditions beneficial for cortical reorganization.

### Size-Dependent Bouton Dynamics

Previous work has focused mostly on bouton turnover, finding variable rates (De Paola et al., 2006, Majewska et al., 2006, Qiao et al., 2016). Persistent boutons are less studied but in aged animals exhibit large fluctuations in size, suggesting reduced synaptic tenacity with aging (Grillo et al., 2013). The activity underpinning such fluctuations is unclear. Our *in vivo* functional experiments combined with simulations suggest that a reduction in the range of correlated network activity shapes the distribution of persistent bouton sizes in deprived cortex, possibly because the reduced range of correlated activity no longer constrains or reinforces synapse strength. Size-dependent dynamics have been reported in a variety of preparations and may represent a general property of synapses (Minerbi et al., 2009, Loewenstein et al., 2011, Statman et al., 2014). Yet, few have investigated how sensory experience influences size-dependent dynamics. We find size-dependent dynamics to be most strongly expressed after deprivation, but we do not exclude the possibility that similar more subtle dynamics occur in naive animals (Loewenstein et al., 2011). Size/strength-dependent plasticity rules are widely used in theoretical models of network function (Kalantzis and Shouval, 2009, van Rossum et al., 2012), and they have been demonstrated *ex vivo* (Sáez and Friedlander, 2009). However, understanding of the *in vivo* biological mechanisms is limited.

### Reduced Range of Network Correlations

We measured cortical activity with chronic calcium imaging, and we estimated the degree of correlated network activity with population coupling values (Okun et al., 2015). Population coupling reflects the strength of a neuron’s relationship to the mean population activity, and it has been shown to correlate with the probability of local input in V1 (Okun et al., 2015). We found strongly coupled neurons became more weakly coupled after deprivation while weakly coupled neurons showed greater population coupling. Such changes in population coupling could be driven by factors including loss of patterned sensory activity, reduced inhibition (Barnes et al., 2015b), or changes in thalamic drive (Linden et al., 2009).

### Measuring Persistent Boutons

Measuring presynaptic structures *in vivo* is challenging (Canty and De Paola, 2011). We adapted a published approach validated by *in vivo* imaging and ultra-structural analysis (Grillo et al., 2013). Our approach has limitations; for example, very small axonal varicosities may not harbor synapses. To exclude this possibility, we restricted our analysis to varicosities with intensities twice as great as the axon backbone, which always harbors synapses (Grillo et al., 2013). Our analysis is unable to distinguish between single and multi-synaptic boutons, however, only a small proportion of boutons make synaptic contacts with multiple spines (Knott et al., 2006). Finally, we studied boutons from L2/3 and L5 pyramidal neurons as well as thalamocortical axons (Feng et al., 2000), but we do not know the identity of their post-synaptic targets. This is an important limitation with implications for circuit function (Lu et al., 2007, Jiang et al., 2015).

### Consequences for the Network

The distribution of synaptic strengths is thought to encode sensory experience (Stepanyants and Escobar, 2011). Thus, the bouton changes we observe may result in the loss of stored sensory information. This process occurred over days and so information may be gradually degraded. Slow degradation may account for the ability to reinstate previous tuning properties when sensory deprivation is reversed, since a fraction of the prior weight distribution may remain (Rose et al., 2016). Although size-dependent bouton dynamics may result in the loss of information, this process may also carry functional benefit. This is because synapses with less extreme weights are thought to have greater potential to undergo both synaptic strengthening and synaptic weakening (van Rossum et al., 2012). In line with this, we found populations of synapses in deprived cortex underwent greater strengthening and weakening following LTP/LTD induction compared to controls. Therefore, the structural plasticity we describe may lead to a loss of stored sensory information but enhance the flexibility of synapses to adapt to novel non-deprived sensory input.

## Experimental Procedures

Further details and an outline of resources used in this work can be found in the Supplemental Experimental Procedures.

### Animals

Experiments were conducted according to the UK Animals (Scientific Procedures) Act 1986. Male and female mice (post-natal day [P]60–P90) were sex and age matched within experimental groups, and they were housed with littermates on a 12-hr light-dark cycle. *Thy1*-GFP mice were used for structural imaging, and C57BL/6 mice were used for electrophysiology and were injected with AAV2/1-*ef1*α-GCaMP5 for functional imaging.

### Statistics

Statistical analysis was performed in MATLAB or SigmaPlot. Comparisons were made using parametric or non-parametric statistics where appropriate. Correction for multiple testing used either Holm-Sidak or Dunn’s method. Correlations were run using Pearson’s correlation test.

## Supplemental Information

Document S1. Supplemental Experimental Procedures and Tables S1–S4

Document S2. Article plus Supplemental Information
