# Supplementary material for: Size-Dependent Axonal Bouton Dynamics following Visual Deprivation In Vivo
Source: Cell Rep. 2018 Jan 29;22(3):576–84. doi: 10.1016/j.celrep.2017.12.065 (PMC5792425; doi:10.1016/j.celrep.2017.12.065)
Supplement: Document S1. Supplemental Experimental Procedures and Tables S1–S4 [file mmc1.pdf]

Cell Reports, Volume *22*

Supplemental Information

Size-Dependent Axonal Bouton Dynamics following Visual Deprivation *In Vivo*

Rosanna P. Sammons, Claudia Clopath, and Samuel J. Barnes

SUPPLEMENTAL EXPERIMENTAL PROCEDURES

CONTACT FOR REAGENT AND RESOURCE SHARING

Further information and requests for resources and reagents should be directed to and will be fulfilled by the Lead Contact, Samuel Barnes (samuel.barnes@imperial.ac.uk).

EXPERIMENTAL MODEL AND SUBJECT DETAILS

Animals

Experiments were conducted according to the U.K. Animals (Scientific Procedures) Act 1986. Male and female mice (P60 – 90) were sex and age matched within experimental groups and housed with littermates on a 12 hr light-dark cycle. *Thy1*-GFP mice (Feng et al., 2000) were used for structural imaging, C57BL/6 mice for electrophysiology and C57BL/6 mice injected with AAV2/1-*ef1*α-GCaMP5 for functional imaging.

METHOD DETAILS

Animal Surgery

Cranial windows were surgically implanted over the right hemisphere of monocular visual cortex, as described previously (Barnes et al., 2015; Holtmaat et al., 2009). We made a craniotomy in ketamine (0.15 mg/g) /xylazine (0.015 mg/g) anesthetized mice and replaced the skull with a glass cover-slip that was attached to the bone with dental cement. Mice were allowed to recover for at least 21 days after surgery before imaging commenced. For monocular enucleation, we applied lidocaine to the area around the left eye prior to surgical removal of the eye. Monocular enucleation was conducted in adulthood (P60 – 90). Control 'sham-enucleated' mice were given time-matched anaesthesia. For structural imaging, animals were imaged while anaesthetised (under ketamine/xylazine) according to the experimental timeline **(Fig 1A)**. For functional imaging experiments, mice were injected with AAV2/1-*ef1*α-GCaMP5 before the glass cover slip was positioned and allowed to recover for 18 days prior to imaging experiments.

Structural imaging

Structural imaging was performed as described previously (Barnes et al., 2015). Briefly, we used a 2-P microscope with a MaiTai BB laser with a DeepSee prechirp unit (Newport/Spectra Physics) set to 909 nm and an Olympus 40× 0.8 NA water immersion objective. The average laser power was < 50 mW as measured at the back focal plane and was kept constant across imaging sessions. For image acquisition, we used Scanimage freeware (VidrioTech). Image acquisition parameters were: 64 × 64 µm, 512 × 512 pixels, 0.5 µm step in depth.

Functional imaging

Functional imaging was performed as described previously (Barnes et al., 2015; Keck et al., 2013; Keller et al., 2012) and data are taken from a previously published data set that was shared (Barnes et al., 2015). We performed different analyses on these data. In brief, functional calcium imaging was performed with a custom built 2-P microscope. The illumination source was a MaiTai eHP laser with a DeepSee prechirp unit (Newport/Spectra Physics) or a Chameleon Vision S (Coherent). The excitation wavelength was set to 910 nm. The scanhead was based on an 8 kHz resonant scanner (Cambridge Technology), used in bidirectional mode. This enabled frame rates of 40 Hz at 400 × 600 pixels. A high power objective Z-piezo stage (Physik Instrumente) was used in order to move the objective down in steps of approximately 20 µm between frames and return to the initial position after four frames. Data were acquired at four different depths, reducing the effective frame rate from 40 Hz to 10 Hz. Laser power was < 50 mW. We used a Nikon 16× 0.8 NA objective. Data were acquired with a 250 MHz digitizer (National Instruments) and pre-processed with a custom programmed FPGA (National Instruments). Head-fixed animals ran on a spherical treadmill while visual stimuli were presented on two screens arranged at 60 degrees relative to each other in front of the mouse. This arrangement is intended to simulate visual flow similar to that experienced when running between two walls. Visual stimuli were full field gratings, the motion of which was controlled by rotation of the spherical treadmill. Experiments consisted of alternating 3 minute blocks in which the mouse either received coupled visual

feedback or the screens were switched off. Each condition was repeated twice, followed by drifting gratings in eight directions (0-360 degrees by 45 degree steps) presented in a random order, with a spatial frequency of 0.04 cycles/degree and a temporal frequency of 2 Hz. The same visual stimulus paradigm was used at each time point before and after enucleation.

Network Simulation

The parameters for the model were kept fixed throughout all simulations and were taken from previously published work (Clopath et al., 2010; Ko et al., 2013). In order to train the network to the 'control' case, feedforward weights were initialized with receptive fields (weights taken from previous test simulations took values between 0 and 3 which were also the hard bounds). At the beginning of the simulation, recurrent excitatory connection weights were set to 1. The network settles in a configuration where neurons receiving correlated inputs develop strong inter-connectivity (Clopath et al., 2010; Ko et al., 2013). For example, in **Fig 3Bi,** neurons 2&3, neurons 4,5&6, and neurons 7&8, 9&10 receive correlated inputs indicated by the fact that they have strong feedforward weights to the same inputs, strong bidirectional lateral connectivity within their group and weak connections outside of their cluster. Note that neurons select their input preference randomly and that neurons were relabelled so that neighbouring neuron numbers are correlated. The model does not take into account physical space. In order to simulate the deprivation paradigm we reduced the range of correlated inputs to the network by replacing highly positively and highly negatively correlated inputs with uncorrelated inputs of the same strength so that neurons in the deprived simulation received the same statistics of input current as well as exhibited the same statistics of spiking as neurons in the control simulation. In particular, we kept the same feedforward weights and the same Poisson firing rate for the inputs as the control condition but every 100 ms, we randomly reallocated the inputs to half of the neurons.

For simulations of network plasticity, we injected input patterns changing every 100 ms and recorded the total positive weight change and the total negative weight change over 1000 s. The simulations were run 50 times. In order to simulate the time it takes for the network to learn a new pattern we removed the inputs to the network (either the control or the deprived network) and replaced them with other correlated inputs of the same strength and correlation level as the control case (e.g. instead of in **Fig 3Bi** neurons 2&3, 4,5&6, and 7&8, 9&10 being correlated, we chose another random assignment such as 2&4, 6&1, 3&10, 5,7&8). We then measured the time it took for the network to learn the new pattern. In particular, we measured how long it took for the lateral weights to become strong within their correlated groups (more than two thirds of their maximal size) and weak across groups (less than a third of the maximal size). The simulations were run 50 times. In a separate simulation we lowered activity in the deprived case rather than reducing the range of correlated network activity. In this alternate model, we found that both weak (0.67 ± 0.10, Normalized Δ in weights, $p = 0.002$, paired t-test) and strong (0.67 ± 0.03, Normalized Δ in weights, $p < 0.001$, paired t-test) synaptic weights decreased in strength.

Electrophysiology

Electrophysiological recordings were conducted as previously described (Barnes et al., 2015). Deeply anaesthetized mice were transcardially perfused with 10 ml of ice-cold (4 ºC) dissection artificial cerebral spinal fluid (ASCF; in mM, 108 choline-Cl, 3 KCl, 26 $NaHCO_3$, 1.25 $NaHPO_4$, 25 D-glucose, 3 Na pyruvate, 2 $CaCl_2$ and 1 $MgCl_2$ saturated with 95% O_2 / 5% CO_2). Coronal brain slices 300 µm thick were cut (Vibratome 3000, Leica) from visual cortex. Slices were incubated for at least 60 minutes in a holding chamber and then recordings were made at room temperature (24 ºC) in a recording ASCF (in mM, 120 NaCl, 3 KCl, 23 $NaHCO_3$, 1.25 $NaHPO_4$, 10 D-glucose, 2 $CaCl_2$ and 1 $MgCl_2$ saturated with 95% O_2 / 5% CO_2). Recordings were targeted to V1m based on stereotaxic coordinates. We recorded from L2/3 on a custom-built microscope under infrared differential interference contrast microscopy. L2/3 pyramidal neurons were identified based on spiking properties and pyramidal shaped soma and recorded in current clamp mode (Multiclamp 700B, Molecular Devices), using Ephus acquisition software (VidrioTech). We did not correct for the liquid junction potential. Patch pipettes (4 – 7 MΩ) contained the following in mM: 130 $KMeSO_3$, 8 NaCl, 2 KH_2PO_4, 2 D-glucose and 10 HEPES. The paired pulse ratio (PPR) values of L2/3 pyramidal neurons were assessed in whole-cell mode using stimulation of horizontal L2/3 inputs with a concentric bipolar stimulating electrode. Extracellular

stimulation at 1 – 10 Hz consisted of 1 ms current pulses with a stimulation intensity set to 50 % of the intensity that evoked the maximum EPSP amplitude. PPR values at four frequencies (1, 2, 5 and 10 Hz) were taken as the average of 20 trials per neuron. LTP and LTD were induced using an adapted method (Boudkkazi et al., 2007). LTD was induced by a low frequency stimulation protocol in which the stimulation was applied at 3 Hz for 3 – 5 minutes whilst the post-synaptic neuron was held at a membrane potential of -40 mV. LTP was induced by stimulating at 1 Hz during 2-3 minutes whilst the postsynaptic neuron was held at -10 mV. All Electrophysiology data was analysed by custom written software in Matlab.

QUANTIFICATION AND STATISTICAL ANALYSIS

Structural imaging Analysis

In vivo 2P images were processed in ImageJ (US National Institutes of Health, Bethesda, MD) and custom written software in Matlab (Mathworks). Fluorescence intensity profiles of axonal stretches including boutons were measured at multiple depths in ImageJ and the three maximum intensity profiles were averaged for each bouton **(Fig 1B)**. The average fluorescence intensity profile was then processed by semi-automated custom software in Matlab. The baseline axonal backbone was binned according to fluorescence intensity and fitted with a linear function and then subtracted to leave the average bouton intensity profile. Individual bouton profiles were then fitted with Gaussian curves and the area under the curve was normalised to the intensity of the axonal backbone so that raw bouton size values in Figure 1 are given in axonal backbone units (unless otherwise stated). All boutons were checked against the original images by two experimenters who were blind to the experimental condition to ensure that boutons were reliably identified as, persistent, disappearing or new. To be included in the analysis boutons had to be at least twice as bright as the background in at least one session, this criteria is based on previous correlative EM and *in vivo* imaging findings which adopted similar normalisation techniques (Grillo et al., 2013). Boutons were defined as persistent if they were present in all imaging timepoints. We sampled similar numbers of boutons per mouse in deprived (43 ± 7, n = 8 mice) and control (42 ± 7, n = 8 mice, p = 0.911, t-test) animals. Furthermore, the average length of axon sampled per mouse was also similar between deprived (22 ± 2, n = 8 mice) and control (24 ± 1, n = 8 mice, p = 0.162, t-test) animals. The initial (0 d) size of the bouton in axonal backbone units was used to define boutons as either small, mid-range or large. Boutons were considered small if their initial size was in the lowest 30th percentile of bouton sizes; large boutons were defined as those whose initial size was in the top 70th to 100th percentile. Those boutons whose size lay between the 30th and 70th percentiles were classified as mid-range. The average bouton size was similar prior to deprivation in control and deprived cortex **(Fig 1Si)**. The bouton turnover ratio (TOR) was calculated using previously published criteria where the TOR between two imaging sessions was defined as (nNew + nDisp)/2N, where nNew and nDisp are the number of new and disappearing boutons respectively and N is the total number of boutons in a session (Grillo et al., 2013).

Functional imaging and analysis

Processing of raw functional imaging data was as described previously (Barnes et al., 2015; Keck et al., 2013) For functional data analysis cells were selected based on mean and maximum projections of the data by hand (the nucleus was excluded from the selection) and high resolution depth stacks were used to ensure that cells did not have a filled nucleus. Fluorescence traces were calculated as the average fluorescence of pixels lying within the cell in each frame. To remove slow signal changes in raw fluorescence traces, the 8th-percentile value of the fluorescence distribution in a ± 15 second window was subtracted from the raw fluorescence signal (Dombeck et al., 2007). Calcium signals (%$\Delta F/F_0$) were calculated by dividing the raw fluorescence signal by the median of each cell's fluorescence distribution. Cellular activity was calculated using the integrated fluorescence as describe previously (Barnes et al., 2015) with a 15 % $\Delta F/F_0$ threshold. Activity was then normalized to the duration of our imaging paradigm to give values in %$\Delta F/F_0$/second. For each imaged region we calculated the mean and the variance of the activity values. Measurements of population coupling were estimated using the 'Pearson Coupling' approach (Okun et al., 2015). We first generated an average population rate trace for each neuron by taking the average fluorescence signal at each time bin for all neurons in a region (excluding the neuron of interest). We then ran Pearson pairwise correlations between the average population rate trace and each neurons activity trace in an imaged region. For each imaged region we calculated the mean and the variance of the population coupling scores. In order to normalize values and pool data across experiments we

took the difference (or absolute difference) between each neurons' population coupling score and the average population coupling score in that imaged region.

Statistics

Statistical analyses were performed either in Matlab or SigmaPlot. Data were tested for equal variance and normality (Shapiro-Wilk test) and then comparisons were made using parametric or non-parametric tests, as appropriate (t-test, paired t-test, Chi-square test, Mann-Whitney Rank Sum test, One-Way ANOVA with Holm-Sidak post-hoc test, Repeated measures ANOVA with Holm-Sidak post-hoc test, or a Two-Way ANOVA with Holm-Sidak post-hoc test). For normalized axonal bouton size, data were $\log_{10}$ transformed (Loewenstein et al., 2011) as noted in the text. Statistical tests were two-sided. Correlation coefficients were calculated with Pearson's correlation coefficient. A power analysis was performed to ensure we used a sufficient sample size. Specific statistical tests used for all figures along with the number of samples and details of center and dispersion measures can be found in supplemental tables S1-S4.

DATA AND SOFTWARE AVAILABILITY

Requests for data and software should be directed to the Lead Contact, Samuel Barnes (samuel.barnes@imperial.ac.uk) and will be made available upon reasonable request.

Code availability

The code used for image registration and data acquisition of the functional data is available at: http://sourceforge.net/projects/iris-scanning/

RESOURCES TABLE

REAGENT or RESOURCE	SOURCE	IDENTIFIER
Bacterial and Virus Strains		
AAV2/1-*ef1*α-GCaMP5	FMI Vector Core	N/A
Chemicals, Peptides, and Recombinant Proteins		
Isoflurane (Attane)	Provet	CAS 26675-46-7
Dental Cement (Paladur)	Heraeus Kulzer	CAS 9066-86-8
Ketamine	Pfizer	CAS 1867-66-9
Xylazine	Rompun	CAS 7361-61-7
Emla Cream 5 %	AstraZeneca	CAS 137-58-6<br>CAS 721-50-6
D Glucose	Sigma-Aldrich	CAS 50-99-7
NaCl,	Tocris	CAS 7647-14-5
KCl	Tocris	CAS 7447-40-7
$NaHCO_3$	Tocris	CAS 144-55-8
NaH_2PO_4	Tocris	CAS 7558-80-7
$CaCl_2$	Sigma-Aldrich	CAS 10043-52-4
$MgSO_4$	Sigma-Aldrich	CAS 7487-88-9
$KMeSO_3$	Sigma-Aldrich	CAS 562-54-9
KH_2PO_4	Sigma-Aldrich	CAS 7778-77-0
Na pyruvate	Sigma-Aldrich	CAS 113-24-6
Choline-Cl	Sigma-Aldrich	CAS 67-48-1
HEPES	Sigma-Aldrich	CAS 7365-45-9
Experimental Models: Organisms/Strains		
Mouse: Thy-1 GFP-M line	JAX	RRID:IMSR_JAX:007788
Mouse: C57BL6/J	Charles River Laboratories	N/A
Software and Algorithms		
Matlab	The MathWorks, Inc.	RRID: SCR_001622

LabView	National Instruments	RRID: SCR_014325
Sigmaplot13	Systat Software, Inc.	N/A
Scanimage	Vidrio technolgies	RRID: SCR_014307
Ephus	Vidrio technolgies	N/A
ImageJ	NIH	RRID: SCR_003070

REFERENCES

Barnes, S.J., Sammons, R.P., Jacobsen, R.I., Mackie, J., Keller, G.B., and Keck, T. (2015). Subnetwork-Specific Homeostatic Plasticity in Mouse Visual Cortex In Vivo. Neuron *86*, 1290–1303.

Boudkkazi, S., Carlier, E., Ankri, N., Caillard, O., Giraud, P., Fronzaroli-Molinieres, L., and Debanne, D. (2007). Release-dependent variations in synaptic latency: a putative code for short- and long-term synaptic dynamics. Neuron *56*, 1048–1060.

Clopath, C., Büsing, L., Vasilaki, E., and Gerstner, W. (2010). Connectivity reflects coding: a model of voltage-based STDP with homeostasis. Nat. Neurosci. *13*, 344–352.

Dombeck, D.A., Khabbaz, A.N., Collman, F., Adelman, T.L., and Tank, D.W. (2007). Imaging large-scale neural activity with cellular resolution in awake, mobile mice. Neuron *56*, 43–57.

Feng, G., Mellor, R.H., Bernstein, M., Keller-Peck, C., Nguyen, Q.T., Wallace, M., Nerbonne, J.M., Lichtman, J.W., and Sanes, J.R. (2000). Imaging neuronal subsets in transgenic mice expressing multiple spectral variants of GFP. Neuron *28*, 41–51.

Grillo, F.W., Song, S., Teles-Grilo Ruivo, L.M., Huang, L., Gao, G., Knott, G.W., Maco, B., Ferretti, V., Thompson, D., Little, G.E., et al. (2013). Increased axonal bouton dynamics in the aging mouse cortex. Proc. Natl. Acad. Sci. U. S. A. *110*, E1514-1523.

Holtmaat, A., Bonhoeffer, T., Chow, D.K., Chuckowree, J., De Paola, V., Hofer, S.B., Hübener, M., Keck, T., Knott, G., Lee, W.-C.A., et al. (2009). Long-term, high-resolution imaging in the mouse neocortex through a chronic cranial window. Nat. Protoc. *4*, 1128–1144.

Keck, T., Keller, G.B., Jacobsen, R.I., Eysel, U.T., Bonhoeffer, T., and Hübener, M. (2013). Synaptic scaling and homeostatic plasticity in the mouse visual cortex in vivo. Neuron *80*, 327–334.

Keller, G.B., Bonhoeffer, T., and Hübener, M. (2012). Sensorimotor mismatch signals in primary visual cortex of the behaving mouse. Neuron *74*, 809–815.

Ko, H., Cossell, L., Baragli, C., Antolik, J., Clopath, C., Hofer, S.B., and Mrsic-Flogel, T.D. (2013). The emergence of functional microcircuits in visual cortex. Nature *496*, 96–100.

Loewenstein, Y., Kuras, A., and Rumpel, S. (2011). Multiplicative dynamics underlie the emergence of the log-normal distribution of spine sizes in the neocortex in vivo. J. Neurosci. Off. J. Soc. Neurosci. *31*, 9481–9488.

Okun, M., Steinmetz, N., Cossell, L., Iacaruso, M.F., Ko, H., Barthó, P., Moore, T., Hofer, S.B., Mrsic-Flogel, T.D., Carandini, M., et al. (2015). Diverse coupling of neurons to populations in sensory cortex. Nature *521*, 511–515.

Panel	Comparison		Test	Control	Deprived	p value	n value
**1C**	Axon software vs Summed volume		Linear Regression	$R^2 = 0.93$		$p < 0.001$	n = 19 Boutons
**1C Inset**	Δ axonal backbone units over time 0-0.5 vs 0-6.0 hrs		t-test	0-0.5 hrs 2.2 ± 0.3 vs 0-6.0 hrs 11.1 ± 2.0		$p < 0.001$	n = 19 Boutons
**1D**	Bouton Turnover Ratio<br><br>Control vs Deprived	3 d	Chi-square test	0.08	0.12	p = 0.017	Control = 55 axons<br><br>Deprived = 62 axons
		6 d		0.05	0.06	p = 0.439	
		9 d		0.03	0.04	p = 0.190	
		16 d		0.06	0.04	p = 0.272	
**1D Inset**	Disappearing Boutons (Control vs Deprived)		Chi-square test	11 %	17 %	p = 0.010	Control = 55 axons<br><br>Deprived = 62 axons
	New Boutons (Control vs Deprived)		Chi-square test	5 %	7 %	p = 0.235	
**1E**	Bouton size distribution		Log normal fit		$R^2 = 0.90$	$p < 0.001$	Deprived =345 Boutons
**1F**	Log transformed bouton size distribution		Gaussian fit		$R^2 = 0.96$	$p < 0.001$	
**1G**	Average of persistent bouton sizes (Log_{10}) per region (vs 0 d)	0 d	One-Way Repeated measures ANOVA	1.46 ± 0.07	1.52 ± 0.05	Deprived p = 0.074<br><br>Control p = 0.908	Control = 11 regions<br><br>Deprived = 10 regions
		3 d		1.47 ± 0.08	1.47 ± 0.05		
		6 d		1.46 ± 0.08	1.41 ± 0.04		
		9 d		1.49 ± 0.09	1.50 ± 0.05		
		16 d		1.48 ± 0.07	1.50 ± 0.04		
**1H**	Variance of persistent bouton sizes (Log_{10}) per region (vs 0 d)	0 d	One-Way Repeated measures ANOVA	0.099 ± 0.014	0.110 ± 0.007	Deprived 0 vs 3 d p = 0.019<br><br>0 vs 6 d p = 0.031<br><br>0 vs 9 d p = 0.007<br><br>0 vs 16 d p = 0.004<br><br>Control p = 0.908	
		3 d		0.110 ± 0.017	0.086 ± 0.008		
		6 d		0.111 ± 0.018	0.085 ± 0.004		
		9 d		0.116 ± 0.017	0.078 ± 0.006		
		16 d		0.110 ± 0.014	0.076 ± 0.004		

Statistical Comparisons for Figure 1 I – O				Group (Center and dispersion or fit)		Result	
**Panel**	**Comparison**		**Test**	**Control**	**Deprived**	**p value**	**n value**
**1I**	Log transformed bouton size distribution		Gaussian fit		R^2 = 0.96	p < 0.001	
**1K**				R^2 = 0.93		p < 0.001	
**1J**	Difference in bouton size distributions (%age count)		Descriptive		< 25th = -8 %<br>25th to 75th = +9 %<br>> 75th = -12 %	N/A	
**1L**				< 25th = +1 %<br>25th to 75th = -8 %<br>> 75th = +1 %			
**1L inset**	Absolute Δ of each bouton from population mean of Log_{10} bouton distribution		One-Way ANOVA	Baseline (0 d)<br>0.27 ± 0.01<br>vs<br>Control (16 d)<br>0.29 ± 0.02	Baseline (0 d)<br>0.27 ± 0.01<br>vs<br>Deprived (16 d)<br>0.21 ± 0.01	Deprived<br>p = 0.007<br><br>Control<br>p = 0.337	
**1M**	Change in bouton size normalized to 0 d<br><br>For boutons of different sizes		Two-Way ANOVA	Small<br>1.07 ± 0.04	Small<br>1.26 ± 0.06	p < 0.001	Control = 343 Boutons<br><br>Deprived = 345 Boutons
				Medium<br>1.02 ± 0.02	Medium<br>1.00 ± 0.03	p = 0.688	
				Large<br>0.95 ± 0.02	Large<br>0.86 ± 0.02	p = 0.045	
**1N**	Average size of large boutons over time<br>(vs 0 d)<br><br>(Normalized axonal backbone units)	0 d	One-Way Repeated measures ANOVA	77 ± 5	69 ± 3	Deprived<br>0 vs 3 d<br>p < 0.001<br><br>0 vs 6 d<br>p < 0.001<br><br>0 vs 9 d<br>p < 0.001<br><br>0 vs 16 d<br>p < 0.001<br><br>Control<br>p = 0.161	
		3 d		68 ± 6	53 ± 4		
		6 d		65 ± 7	42 ± 3		
		9 d		72 ± 6	48 ± 3		
		16 d		65 ± 5	43 ± 3		
**1O**	Average size of small boutons over time<br>(vs 0 d)<br><br>(Normalized axonal backbone units)	0 d	One-Way Repeated measures ANOVA	16 ± 1	17 ± 1	Deprived<br>0 vs 3 d<br>p = 0.002<br><br>0 vs 6 d<br>p = 0.007<br><br>0 vs 9 d<br>p < 0.001<br><br>0 vs 16 d<br>p < 0.001<br><br>Control<br>p = 0.154	
		3 d		18 ± 2	25 ± 2		
		6 d		18 ± 1	24 ± 2		
		9 d		17 ± 1	32 ± 3		
		16 d		20 ± 1	33 ± 3		

Statistical Comparisons<br>for Figure 1 P – S			Group<br>(Center and dispersion or fit)		Result	
**Panel**	**Comparison**	**Test**	**Control**	**Deprived**	**p value**	**n value**
**1P**	Comparison of sizes at 16 d to medium size at 16 d<br>(sizes were initially binned based on baseline values 0 d)	One-Way ANOVA	Small (16 d)<br>20 ± 2<br>vs<br>Medium (16 d)<br>37 ± 3	Small (16 d)<br>31 ± 2<br>vs<br>Medium (16 d)<br>35 ± 4	Deprived<br>p = 0.778<br><br>Control<br>p = 0.003	Control = 343 Boutons<br><br>Deprived = 345 Boutons
			Large (16 d)<br>65 ± 5<br>vs<br>Medium (16 d)<br>37 ± 3	Large (16 d)<br>43 ± 3<br>vs<br>Medium (16 d)<br>35 ± 4	Deprived<br>p = 0.106<br><br>Control<br>p < 0.001	
**1Q**	Bouton size vs rate of change between imaging sessions	Pearson Correlation		r = -0.51	p < 0.001	
			r = -0.23		p < 0.001	
**1R**	Rate of change<br>0 – 3 d vs 9 – 16 d<br>(absolute rate of change in bouton size Log$_{10}$/day)	MWRST		0 – 3 d<br>0.07, 0.03 – 0.10<br>vs<br>9 – 16 d<br>0.04, 0.01 – 0.07	Deprived<br>p < 0.001	
**1Si**	Bouton size (0 d)<br><br>(Normalized axonal backbone units)	t-test	43 ± 3	42 ± 2	p = 0.725	
**1Sii**	Bouton variance (0 d)<br><br>(Normalized axonal backbone units)		22 ± 3	22 ± 2	p = 0.893	Control = 11 regions<br><br>Deprived = 10 regions
Control = 343 boutons from 55 axons measured in 8 mice (42 ± 7 boutons/mouse)<br>Deprived = 345 boutons from 62 axons measured in 8 mice (43 ± 7 boutons/mouse)						

Table S1. Statistical comparisons for Figure 1, Related to Figure 1.

Statistical Comparisons for Figure 2 B – K			Group (Center and dispersion or fit)		Result	
**Panel**	**Comparison**	**Test**	**Control**	**Deprived**	**p value**	**n value**
**2B**	Average activity per region compared to baseline (Normalized)	One-Way Repeated Measures ANOVA	0 d<br>1.00 ± 0.02<br>vs<br>3 d<br>0.99 ± 0.02	0 d<br>1.00 ± 0.02<br>vs<br>3 d<br>0.94 ± 0.02	p = 0.144	
**2B**	Average activity per region Control vs Deprived (Normalized)	t-test	3 d<br>0.99 ± 0.02	3 d<br>0.94 ± 0.02	p = 0.141	
**2C**	Variance of activity per region compared to baseline (Normalized)	One-Way Repeated Measures ANOVA	0d<br>1.00 ± 0.04<br>vs<br>3 d<br>1.15 ± 0.04	0 d<br>1.00 ± 0.06<br>vs<br>3 d<br>1.05 ± 0.05	p = 0.096	Deprived<br>n = 32 regions<br><br>Control<br>n = 32 regions
**2C**	Variance of activity per region Control vs Deprived (Normalized)	t-test	3 d<br>1.15 ± 0.04	3 d<br>1.05 ± 0.05	p = 0.144	
**2E**	Mean population coupling scores per region compared to baseline (Normalized)	One-Way Repeated Measures ANOVA	0 d<br>1.00 ± 0.03<br>vs<br>3 d<br>1.03 ± 0.03	0 d<br>1.00 ± 0.03<br>vs<br>3 d<br>1.05 ± 0.03	p = 0.469	
**2E**	Mean population coupling scores per region Control vs Deprived (Normalized)	t-test	3 d<br>1.03 ± 0.03	3 d<br>1.05 ± 0.03	p = 0.660	
**2F**	Variance of population coupling scores per region compared to baseline (Normalized)	One-Way Repeated measures ANOVA	0 d<br>1.00 ± 0.07<br>vs<br>3 d<br>0.92 ± 0.06	0 d<br>1.00 ± 0.07<br>vs<br>3 d<br>0.76 ± 0.04	Deprived<br>p = 0.017<br><br>Control<br>p = 0.534	
**2F**	Variance of population coupling scores per region Control vs Deprived (Normalized)	t-test	3 d<br>0.92 ± 0.06	3 d<br>0.76 ± 0.04	p = 0.033	
**2I**	Difference in normalized population coupling distributions	Descriptive		< 25th = -7 %<br>25th to 75th = +11 %<br>> 75th = -6 %	N/A	
**2J**	Absolute difference from population coupling mean (0 vs 3 d)	MWRST		0 d<br>0.15, 0.08 – 0.22<br>vs<br>3 d<br>0.12, 0.06 – 0.19	p < 0.001	
**2K**			0 d<br>0.13, 0.07 – 0.19<br>vs<br>3 d<br>0.13, 0.07 – 0.18		p = 0.248	

**Statistical Comparisons for Figure 2 L – M**			**Group (Center and dispersion or fit)**		**Result**	
**Panel**	**Comparison**	**Test**	**Control**	**Deprived**	**p value**	**n value**
**2L**	Weakly coupled Neurons (-0.5 to -0.1) compared to baseline (0 vs 3 d)	Paired t-test		0 d -0.184 ± 0.003 vs 3 d -0.059 ± 0.007	$p < 0.001$	Deprived n = 32 regions Control n = 32 regions
**2L**	Strongly coupled Neurons (+0.2 to +0.5) compared to baseline (0 vs 3 d)	Paired t-test		0 d 0.323 ± 0.006 vs 3 d 0.082 ± 0.019	$p < 0.001$	
**2M**	Weakly coupled Neurons Control vs Deprived (3 d)	t-test	3 d -0.101 ± 0.007	3 d -0.059 ± 0.007	$p < 0.001$	
**2M**	Strongly coupled Neurons Control vs Deprived (3 d)	t-test	3 d 0.184 ± 0.015	3 d 0.082 ± 0.019	$p < 0.001$	
Deprived = 1081 neurons from 32 regions in 4 animals<br>Control = 1155 neurons from 32 regions in 4 animals						

Table S2. Statistical comparisons for Figure 2, Related to Figure 2.

Statistical Comparisons for Figure 3 D – M			Group (Center and dispersion or fit)		Result	
**Panel**	**Comparison**	**Test**	**Control**	**Deprived**	**p value**	**n value**
**3D**	Δ strength of weak weights	Paired t-test		Timepoint 1 0.23 ± 0.01 vs Timepoint 700 1.11 ± 0.04	p < 0.001	Synaptic weights = 100<br><br>Trials = 100<br><br>700 or 1000 timepoints
**3E**	Δ strength of strong weights	Paired t-test		Timepoint 1 2.97 ± 0.01 vs Timepoint 700 1.40 ± 0.02	p < 0.001	
**3E inset**	Standard deviation of all synaptic weights across stimulation<br><br>Timepoint 1 vs Timepoint 700	Paired t-test		Timepoint 1 0.97 ± 0.01 vs Timepoint 700 0.91 ± 0.02	p < 0.001	
**3G**	Increase in synaptic weights following positively correlated bursts	t-test	0.125 ± 0.006	0.192 ± 0.009	p < 0.001	Synaptic weights = 100<br><br>Input trials = 50
**3H**	Decrease in synaptic weights following negatively correlated bursts	t-test	0.021 ± 0.001	0.035 ± 0.002	p < 0.001	
**3M**	Time to learn novel patterns (seconds x 10^3):	t-test	1.72 ± 0.01	1.45 ± 0.01	p = 0.012	Synaptic weights = 100<br><br>Patterns = 50
Network simulation = 100 synaptic weights, run for 100 trials over 700 or 1000 timepoints.						

Table S3. Statistical comparisons for Figure 3, Related to Figure 3.

Statistical Comparisons for Figure 4 D – I				Group (Center and dispersion or fit)		Result	
**Panel**	**Comparison**		**Test**	**Control**	**Deprived**	**p value**	**n value**
**4D inset**	Average PPR value		t-test	0.86 ± 0.03	0.85 ± 0.02	p = 0.801	Control = 15 neurons<br><br>Deprived = 20 neurons
**4D**	Range of PPR values (absolute Δ from population mean)		MWRST	0.16, 0.07 – 0.23	0.09, 0.05 – 0.21	p = 0.028	
**4E**	LTP Control vs Deprived (Normalized values)	-5 mins	Two-Way ANOVA	1.00 ± 0.03	0.94 ± 0.04	p = 0.507	Control = 21 neurons<br><br>Deprived = 24 neurons
		-2.5 mins		1.00 ± 0.04	1.00 ± 0.04	p = 0.968	
		+2.5 mins		1.12 ± 0.07	1.19 ± 0.07	p = 0.426	
		+5 mins		1.07 ± 0.03	1.37 ± 0.07	p < 0.001	
		+7.5 mins		1.26 ± 0.05	1.58 ± 0.07	p < 0.001	
		+10 mins		1.30 ± 0.07	1.54 ± 0.08	p = 0.005	
		+12.5 mins		1.31 ± 0.05	1.50 ± 0.09	p = 0.029	
		+15 mins		1.44 ± 0.06	1.65 ± 0.08	p = 0.014	
		+17.5 mins		1.39 ± 0.04	1.65 ± 0.10	p = 0.006	
		+20 mins		1.44 ± 0.06	1.69 ± 0.10	p = 0.007	
**4F**	LTD Control vs Deprived (Normalized values)	-5 mins	Two-Way ANOVA	1.00 ± 0.04	0.99 ± 0.03	*p* = 0.827	
		-2.5 mins		1.00 ± 0.03	1.00 ± 0.03	*p* = 0.944	
		+2.5 mins		0.88 ± 0.05	0.73 ± 0.06	*p* = 0.029	
		+5 mins		0.82 ± 0.05	0.70 ± 0.04	*p* = 0.049	
		+7.5 mins		0.88 ± 0.05	0.65 ± 0.04	*p* < 0.001	
		+10 mins		0.89 ± 0.06	0.67 ± 0.04	*p* < 0.001	
		+12.5 mins		0.89 ± 0.06	0.69 ± 0.04	*p* = 0.003	
		+15 mins		0.85 ± 0.06	0.66 ± 0.04	*p* = 0.003	
		+17.5 mins		0.91 ± 0.05	0.62 ± 0.03	*p* < 0.001	
		+20 mins		0.89 ± 0.06	0.60 ± 0.04	*p* < 0.001	
**4G**	Δ in PPR after LTP (normalized to baseline BL)		Paired t-test	BL 1.00 ± 0.07 vs Post LTP 0.91 ± 0.05		p = 0.032	
**4H**					BL 1.00 ± 0.05 vs Post LTP 0.80 ± 0.04	p = 0.003	
**4G**	Δ in PPR after LTD (normalized to baseline BL)		Paired t-test	BL 1.00 ± 0.06 vs Post LTD 1.12 ± 0.06		p = 0.025	
**4H**					BL 1.00 ± 0.07 vs Post LTD 1.46 ± 0.08	p < 0.001	
**4I**	Δ in PPR after LTP Control vs Deprived On Log10 transformed data		t-test	Post LTP 0.91 ± 0.05	Post LTP 0.80 ± 0.04	p = 0.046	
	Δ in PPR after LTD Control vs Deprived		t-test	Post LTD 1.12 ± 0.06	Post LTD 1.46 ± 0.08	p = 0.002	
Figure 4 B-D uses 15 control and 20 deprived neurons from 8 control and 8 deprived animals.<br>Figure 4 E-I uses 21 control and 24 deprived neurons from 7 control and 7 deprived animals.							

Table S4. Statistical comparisons for Figure 4, Related to Figure 4.
