# Supplementary material for: Size-Dependent Axonal Bouton Dynamics following Visual Deprivation In Vivo
Source: Cell Rep. 2018 Jan 29;22(3):576–84. doi: 10.1016/j.celrep.2017.12.065 (PMC5792425; doi:10.1016/j.celrep.2017.12.065)
Supplement: Document S2. Article plus Supplemental Information [file mmc2.pdf]

Cell Reports

Report

Size-Dependent Axonal Bouton Dynamics following Visual Deprivation *In Vivo*

Graphical Abstract

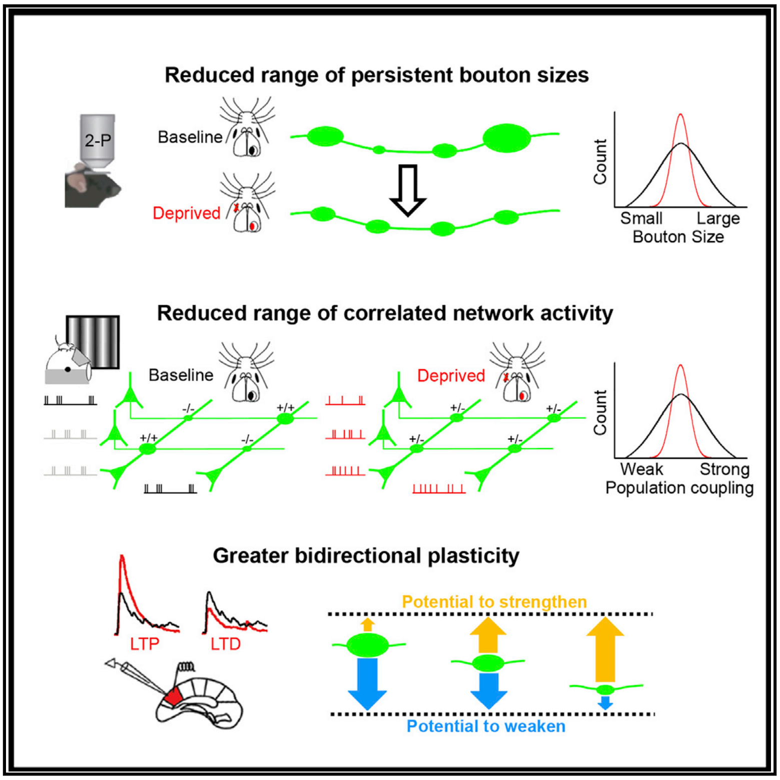

Authors

Rosanna P. Sammons, Claudia Clopath, Samuel J. Barnes

Correspondence

samuel.barnes@imperial.ac.uk

In Brief

Sammons et al. investigate the structural dynamics of persistent axonal boutons. Although central to many theories of sensory processing, persistent synapses remain understudied. Visual deprivation collapses the distribution of bouton sizes toward the mean. Bouton plasticity is accompanied by a reduced range of correlated network activity and greater bidirectional plasticity.

Highlights

- The range of persistent axonal bouton sizes is reduced following visual deprivation
- Bouton sizes move toward the mean in a size-dependent manner
- Bouton plasticity is accompanied by a reduced range of correlated network activity
- Deprived cortex exhibits greater bidirectional functional presynaptic plasticity

Sammons et al., 2018, Cell Reports *22*, 576–584
January 16, 2018 © 2017 The Author(s).
https://doi.org/10.1016/j.celrep.2017.12.065

CellPress

OPEN ACCESS
CellPress

Cell Reports
Report

Size-Dependent Axonal Bouton Dynamics following Visual Deprivation *In Vivo*

Rosanna P. Sammons,[1,4] Claudia Clopath,[2] and Samuel J. Barnes[3,5,*]
[1]Department of Neuroscience, Physiology and Pharmacology, University College London, 21 University St., London WC1E 6DE, UK
[2]Department of Biomedical Engineering, Imperial College London, South Kensington Campus, London SW7 2AZ, UK
[3]Division of Brain Sciences, Department of Medicine, Imperial College London, Hammersmith Hospital Campus, Du Cane Road, London W12 0NN, UK
[4]Present address: Neuroscience Research Centre, Charité Universitätsmedizin Berlin, 10117 Berlin, Germany
[5]Lead Contact
*Correspondence: samuel.barnes@imperial.ac.uk
https://doi.org/10.1016/j.celrep.2017.12.065

SUMMARY

Persistent synapses are thought to underpin the storage of sensory experience, yet little is known about their structural plasticity *in vivo*. We investigated how persistent presynaptic structures respond to the loss of primary sensory input. Using *in vivo* two-photon (2P) imaging, we measured fluctuations in the size of excitatory axonal boutons in L2/3 of adult mouse visual cortex after monocular enucleation. The average size of boutons did not change after deprivation, but the range of bouton sizes was reduced. Large boutons decreased, and small boutons increased. Reduced bouton variance was accompanied by a reduced range of correlated calcium-mediated neural activity in L2/3 of awake animals. Network simulations predicted that size-dependent plasticity may promote conditions of greater bidirectional plasticity. These predictions were supported by electrophysiological measures of short- and long-term plasticity. We propose size-dependent dynamics facilitate cortical reorganization by maximizing the potential for bidirectional plasticity.

INTRODUCTION

Sensory experience modifies synaptic strength and connectivity between cortical neurons (Cheetham et al., 2007; Albieri et al., 2015). Theoretical studies suggest that the distribution of synaptic strengths both processes sensory input and provides a substrate to encode experience (Barbour et al., 2007; Stepanyants and Escobar, 2011). If sensory experience is stored in the synaptic strength distribution, then what happens to this distribution when primary sensory input is removed?

Diminished sensory experience can induce synapse loss but its effect on surviving synapses is less clear (Barnes et al., 2015a, 2017). Conditions that drive synapse loss may weaken surviving synapses (Albieri et al., 2015; Barnes et al., 2015a) or trigger homeostatic changes in strength (Barnes et al., 2017). Alternately, prolonged loss of sensory activity may shape the distribution of surviving synaptic strengths. In this scenario, theoretical studies predict the loss of patterned sensory input would narrow the range of synaptic strengths/weights (Barbour et al., 2007). To test between these scenarios, we removed the contralateral visual drive to mouse monocular primary visual cortex (V1m), and we studied the dynamics of persistent presynaptic structures.

Persistent presynaptic structures are understudied. This is surprising given their abundance in the cortex (Qiao et al., 2016). More attention has been paid to transient synaptic structures, which are thought to be critical for circuit reorganization (De Paola et al., 2006; Keck et al., 2008; Barnes and Cheetham, 2014). Persistent synaptic structures exhibit fluctuations in size both *in vivo* (Grillo et al., 2013; Keck et al., 2013; Barnes et al., 2017) and in reduced preparations (Minerbi et al., 2009; Meyer et al., 2014). We used fluctuations in the size of persistent axonal boutons as a proxy for synaptic strength *in vivo* over time (Canty and De Paola, 2011; Grillo et al., 2013). This approach is supported by work showing a strong correlation between the size of a dendritic spine or axonal bouton and its synaptic strength (Murthy et al., 2001; Noguchi et al., 2011; Cheetham et al., 2014).

Here we use *in vivo* two-photon (2P) imaging to measure fluctuations in the size of persistent axonal boutons in L2/3 of adult V1m following monocular enucleation. We find a reduction in the range of bouton sizes in deprived cortex. After deprivation, large boutons shrink and small boutons grow so that the distribution of bouton sizes narrows. These size-dependent dynamics are accompanied by a reduction in the range of correlated network activity in L2/3 of awake animals. Computational modeling, based on our *in vivo* data, suggests that size-dependent dynamics may be driven by a reduced range of correlated network activity. Furthermore, our model suggests that bouton dynamics increase the potential for bidirectional plasticity (i.e., greater potential for both synaptic strengthening and weakening). Electrophysiological experiments support the predictions of our simulation, and they show greater short- and long-term presynaptic plasticity after deprivation. Our results suggest that size-dependent bouton dynamics may generate conditions that benefit reorganization after deprivation by enhancing the flexibility of synapses to undergo both synaptic strengthening and weakening.

 © 2017 The Author(s).
This is an open access article under the CC BY license (http://creativecommons.org/licenses/by/4.0/).

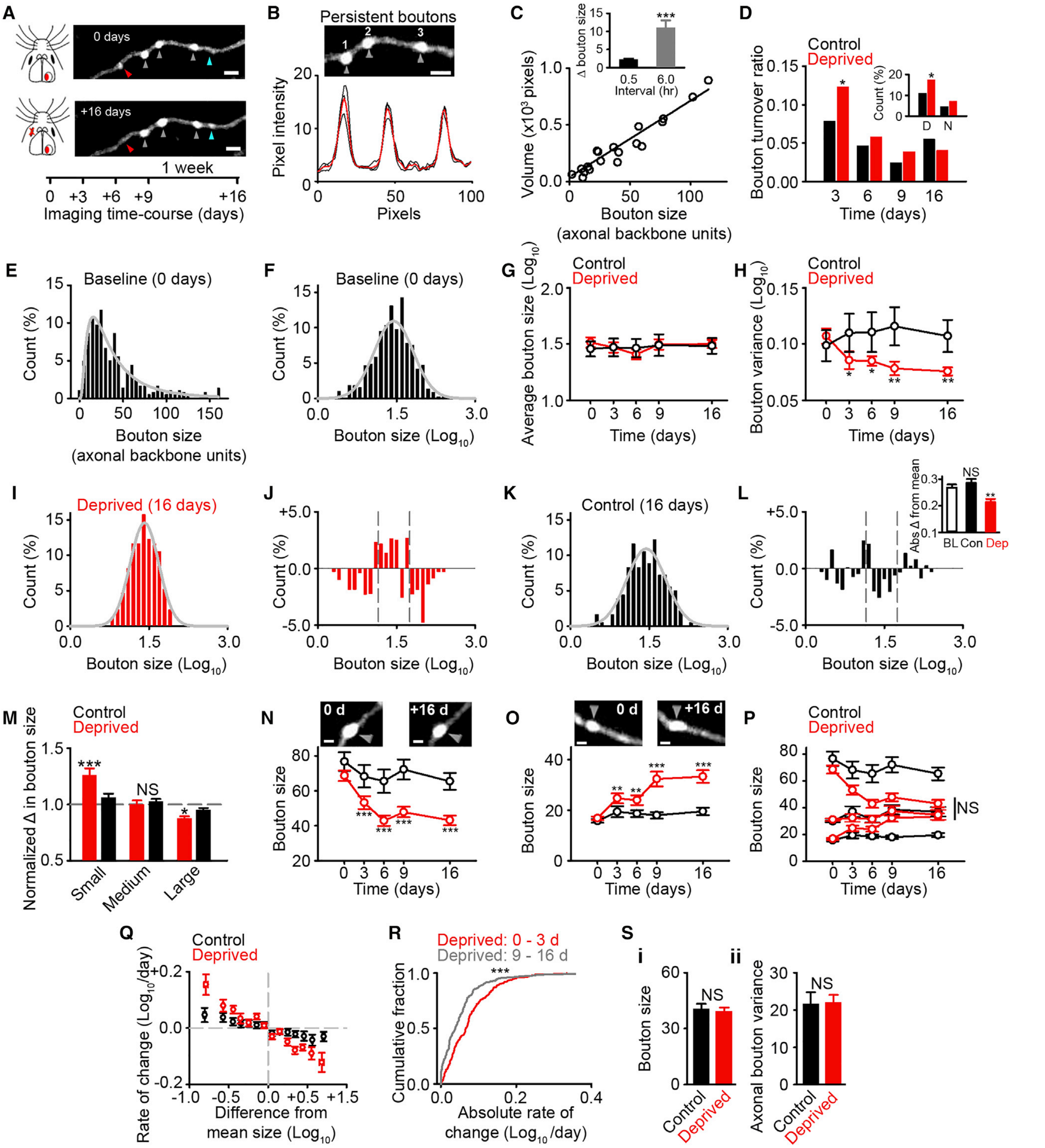

Figure 1. Size-Dependent Bouton Dynamics in Deprived Cortex

(A) Time course of 2P imaging paradigm and enucleation. Axon from L2/3 in V1m of *Thy1*-GFP mouse is shown. Arrows indicate lost (red), new (cyan), and persistent boutons (gray). Scale bar, 2 μm.

(B) Intensity profiles from three persistent boutons above. Red, average taken at 3 depths. Scale bar, 2 μm.

(C) Comparison of analysis program versus summed volume. (Inset) Bouton size over short (0.5 hr) versus longer periods (6 hr) is shown.

(D) Turnover ratios for deprived and control cortex. (Inset) Percentage of disappearing (D) and new (N) boutons at 3 days is shown.

(E) Histogram of bouton size in axonal backbone units (0 day).

(legend continued on next page)

OPEN ACCESS
CellPress

RESULTS

Size-Dependent Bouton Dynamics

We repeatedly imaged axonal boutons through a cranial window implanted over V1m in adult *Thy1*-GFP mice (Feng et al., 2000) before and after monocular enucleation (Figure 1A). Using published criteria, we defined axonal swellings as boutons (Grillo et al., 2013), which were considered persistent if present at each imaging time point (Figure 1A). Fluctuations in persistent bouton size were measured using custom software and quantified as normalized axonal backbone units. Bouton measurements correlated with manual measurements of summed bouton volume (Figures 1B and 1C), and, although stable within imaging sessions, they fluctuated over longer periods (Figure 1C, inset). Measurements of bouton turnover found a small increase 3 days after deprivation (Figure 1D), attributable to more disappearing boutons (Figure 1D, inset). However, after day 3, turnover rates were similar to control values (Figure 1D).

Dendritic spines can undergo homeostatic increases in size following sensory deprivation (Keck et al., 2013; Barnes et al., 2017); whether axonal boutons do too is unclear. We measured the size of persistent boutons following deprivation as a proxy for synaptic strength (Murthy et al., 2001; Cheetham et al., 2014; Grillo et al., 2013). Similar to work investigating dendritic spine sizes *in vivo* (Loewenstein et al., 2011), we found the bouton size distribution during baseline was positively skewed, reminiscent of a log-normal distribution (Figure 1E). A histogram of Log_{10} bouton sizes demonstrated that the bouton size distribution was approximated by a log-normal function (Figure 1F). We compared the average bouton size per imaged region at each time point to baseline, and we found no net shift in average bouton size following deprivation (Figure 1G). Instead, the variance of bouton sizes (per imaged region) was reduced; this was not the case in control cortex (Figure 1H).

A reduction in the size of large boutons without any change in smaller boutons would lead to a reduced range of bouton sizes, as would an increase in the size of smaller boutons with no change in large boutons. Such changes may have important implications, as synapse size influences the potential for synaptic plasticity (Matsuzaki et al., 2004). Thus, we next asked how the bouton size distribution changed after deprivation. We subtracted the deprived distribution (16 days) (Figure 1I) from the baseline distribution (Figure 1F), and we observed fewer large and small boutons in the distribution tails and more boutons around the median (Figures 1I and 1J). This was not the case for time-matched controls (Figures 1K and 1L). To quantify the reduction in bouton variance, we took the absolute difference of each bouton from the mean of the Log_{10} population (Figure 1L, inset). The absolute difference of each bouton was lower in deprived cortex than baseline (Figure 1L, inset). This was not the case for time-matched controls (Figure 1L, inset). We next asked how individual bouton changes narrow the distribution of bouton sizes. We binned boutons by their size before deprivation (see the Supplemental Experimental Procedures), and we examined the change in size over time. We saw a strong inverse correlation between initial bouton size and change in size in deprived cortex (Figure 1M). In contrast, large and small boutons in controls showed similar changes to medium-sized boutons (Figure 1M). The changes in deprived cortex made boutons of different sizes become similar to the mean bouton size (Figures 1N–1P). Large boutons became smaller (Figures 1N and 1P) while small boutons increased in size (Figures 1O and 1P). This was not the case in control cortex for boutons binned identically (Figures 1N–1P).

We next investigated the temporal dynamics of boutons between imaging sessions. We focused on bouton size (measured with respect to the mean on each session) and the change in size expressed in the next session. We found a strong inverse relationship between bouton size and the rate of change in deprived cortex (Figure 1Q). Boutons with the greatest difference from the mean showed the most movement toward the mean on the next session. A weaker correlation was also evident in control cortex (Figure 1Q). Thus, subtle ongoing size-dependent structural dynamics occur in control cortex but are enhanced following deprivation. In deprived cortex, the rate of change slowed in later sessions, as boutons differed less from the mean (Figure 1R). Our results could not be explained by different structural properties prior to deprivation, as axons in deprived cortex had similar bouton size (Figure 1Si) and variance (Figure 1Sii) values to controls. Together our results show the range of bouton sizes is reduced in deprived cortex because boutons regress to the mean at rates proportional to their size. This reduces the difference between boutons that were previously either large (putative strong synapses) or small (putative weak synapses).

Reduced Range of Correlated Network Activity

To estimate network conditions in deprived cortex, we used chronic 2P calcium imaging of GCaMP5 (Akerboom et al., 2012) in L2/3 of V1m in awake mice (Figure 2A). L2/3 excitatory neurons are a putative target and one source (see the Discussion: Measuring Persistent Boutons) of our imaged boutons

(F) Histogram of Log_{10} bouton sizes from (E).
(G) Average persistent bouton size per region in control and deprived cortices.
(H) Variance of persistent axonal boutons per region in control and deprived cortices.
(I and K) Log_{10} bouton sizes in deprived (I, 16 days) and control (K, 16 days) cortices.
(J and L) Difference in deprived (J) and control (L) Log_{10} bouton sizes between 16 days and 0 day. Gray lines, 25th and 75th percentiles. (L, inset) Absolute difference in bouton size from Log_{10} mean at baseline (0 day, black, open), in control cortex (16 days, black, filled) and deprived cortex (16 days, red, filled), is shown.
(M) Normalized change in size of small, medium, and large boutons relative to baseline in control and deprived cortices.
(N–P) Time course of size-dependent bouton dynamics in deprived (red) and control (black) cortices for large (N), small (O), and all boutons (P). Scale bar, 1 μm.
(Q) Rate of change per day between imaging sessions for boutons at different sizes, relative to the mean size, in control and deprived cortices.
(R) Absolute rate of change per day for boutons in deprived cortex between 0 and 3 days or 9 and 16 days.
(S) Persistent bouton size (i) and the variance (ii) of bouton sizes in axonal backbone units in control and deprived cortices. For statistical comparisons and n values, see Table S1. NS, not significant; *p < 0.05, **p < 0.01, and ***p < 0.001. Error bars, mean and SEM.

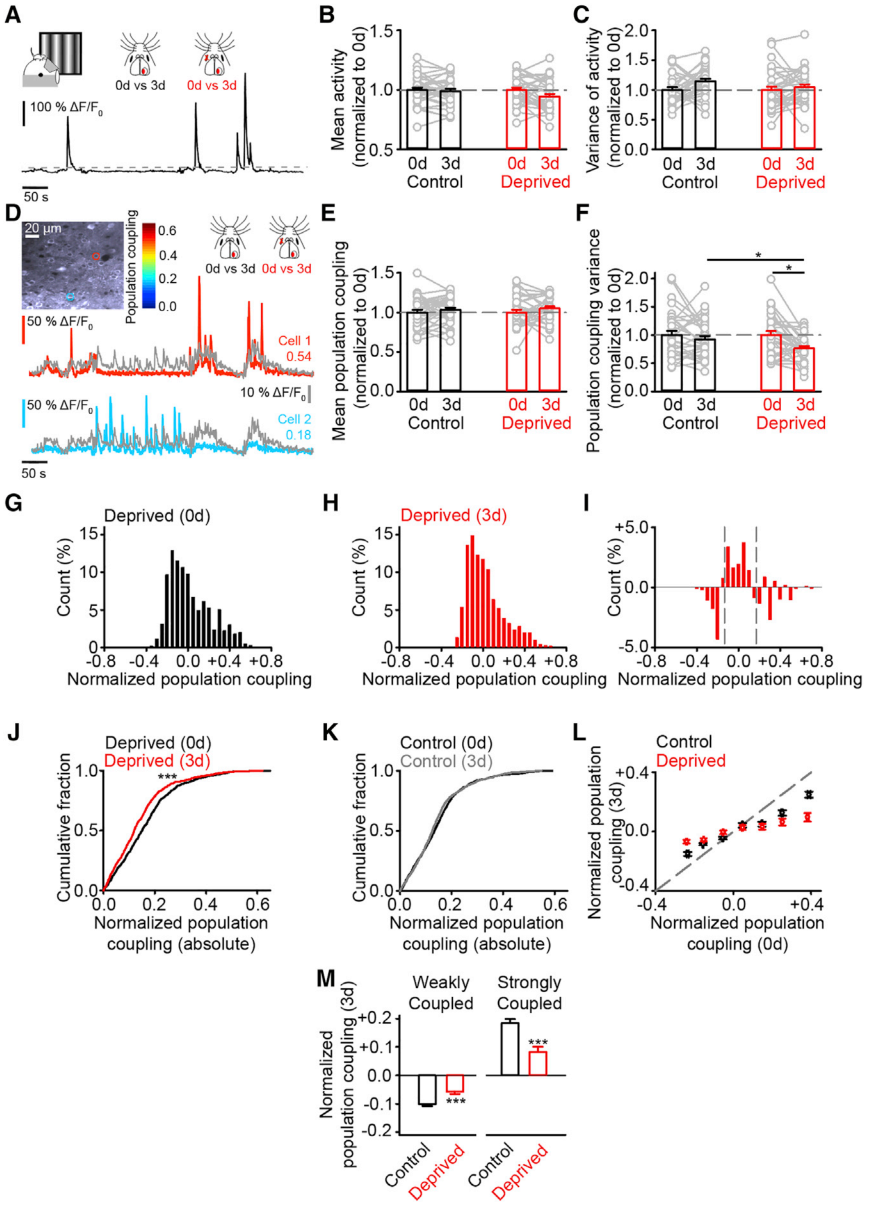

Figure 2. Reduced Range of Correlated Network Activity

(A) Awake behaving imaging of GCaMP5 at 0 day or 3 days with example calcium trace. Gray dashed line is activity threshold. Scale bars, 100% $\Delta F/F_0$ and 50 s.

(B and C) Mean (B) and variance (C) of activity in imaged region normalized to the baseline (0 day) for control and deprived cortices. Gray points show each region.

(D) Example neurons highlighted by regions of interest (ROIs) with color corresponding to population coupling scale bar. GCaMP5 traces show population rate (gray trace), and color-coded traces are according to coupling scores (scores next to traces). Scale bars, individual cells: 50% $\Delta F/F_0$ and 50 s; average population rate trace: 10%$\Delta F/F_0$ and 50 s; region: 20 μm.

(E and F) Mean (E) and variance (F) of population coupling scores per region normalized to the baseline (0 day) for control and deprived cortices. Gray points show each imaged region.

(G and H) Normalized population coupling scores from deprived mice at 0 day (G) and 3 days (H).

(I) Difference between deprived 3 days (from H) and deprived 0 day (from G) normalized population coupling distributions. Gray dashed lines are 25th and 75th percentiles.

(J and K) Absolute normalized population coupling distributions in deprived (J) and control (K) cortices at 0 day and 3 days.

(L) Normalized population coupling scores at 0 day and 3 days for neurons in control and deprived cortices.

(M) Normalized population coupling scores for neurons in control and deprived cortices at 3 days that were either (left) weakly coupled or (right) strongly coupled to population activity when measured at 0 day. For statistical comparisons and n values, see Table S2. *$p < 0.05$ and ***$p < 0.001$. Error bars, mean and SEM.

(Feng et al., 2000), and thus they are a reasonable approximation of network activity for our boutons. Structural imaging data found size-dependent bouton plasticity at 3 days; thus, we compared network activity at day 0 to 3 days. We compared activity between deprived and control mice and within the same mice before and after deprivation (Figure 2A). We measured activity in each region using the integral of the calcium signal for each cell and normalizing this to the duration of imaging (Figure 2A). For both control and deprived animals, we normalized activity levels at all time points to baseline activity. We found the mean and the variance of activity in deprived cortex was similar both to baseline and time-matched control animals (Figures 2B and 2C). These results are consistent with homeostatic recovery of cortical activity following visual deprivation *in vivo* (Barnes et al., 2015b).

To determine how deprivation influences the correlation structure of cortical activity, we used the Pearson coupling statistic to generate population coupling scores for each neuron (Okun et al., 2015). We took the pairwise correlation value between each activity trace and the calcium trace of mean population activity (see the Supplemental Experimental Procedures) (Figure 2D). We normalized to the mean of the baseline, and we examined the mean and variance of population coupling scores in imaged regions over time. Changes in the mean population coupling score in an imaged region (relative to baseline) reflect the strength of correlations between neurons and mean population rate. In contrast, changes in the variance of population coupling scores reflect the range of correlated activity between different neurons and the mean population rate. Mean population coupling scores were similar to both baseline and time-matched controls (Figure 2E). In contrast, the variance of population coupling values in deprived cortex was reduced relative to baseline and control cortex (Figure 2F).

We combined all imaged regions by taking the difference between each neuron's population coupling score in an imaged region and the average population coupling score of that region (see the Supplemental Experimental Procedures). Normalizing in this way allowed us to compare the distributions of population coupling values before (Figure 2G) and after deprivation (Figure 2H). We found fewer normalized population coupling scores in the tails of our distribution and more scores around the median after deprivation (Figure 2I). To quantify these changes, we repeated our normalization approach but instead took the absolute value of the normalized population coupling scores. This approach found the absolute difference from the population coupling mean for each neuron was reduced in deprived (Figure 2J), but not control, cortex (Figure 2K). Examining the behavior of individual neurons found cells with low normalized population coupling scores prior to deprivation had increased scores, relative to baseline and control cortex after deprivation (Figures 2L and 2M). Conversely, neurons with stronger population scores prior to deprivation weakened (Figures 2L and 2M). Thus, mean activity levels and mean population coupling scores were similar in deprived and control cortex, but the range of population coupling scores was reduced after deprivation. This occurred via a reduction in the strength of neurons with strong coupling and an increase in the strength of neurons with weak coupling.

Network Simulation of Strength-Dependent Dynamics

To gain mechanistic insight into size-dependent dynamics, we used a network simulation based on activity measured *in vivo* and spike-timing-dependent plasticity rules (Clopath et al., 2010; Ko et al., 2013). Our simulation had a presynaptic input layer that sent feedforward excitation to postsynaptic neurons (Figures 3A and 3B; see the Supplemental Experimental Procedures). The model's synaptic weights were trained with correlated input. Neurons with positively correlated input had strong connections, and those with negatively correlated input had weak connections (Figure 3Bii). We used the simulated weights to approximate pre-deprivation conditions. Then, in line with measurements of activity, we reduced the range of correlated input (Figure 3C), and we measured changes in weak (Figure 3D) and strong (Figure 3E) weights. Our model found strength-dependent weight dynamics (Figures 3D and 3E) mirrored structural changes *in vivo* (Figures 1N–1P). Reducing the range of correlated activity caused weak weights to strengthen (Figure 3D) and strong weights to weaken (Figure 3E) and, thus, reduced the range of weights (Figure 3E, inset). This occurred because strong weights are maintained by highly positive correlations. Thus, shifting to less correlated activity caused weakening. Similarly, weak weights were driven by negative correlations, so that less negatively correlated activity caused strengthening.

Both our *in vivo* imaging data and our network simulation found size/strength-dependent bouton dynamics resulted in a reduced range of synaptic weights/bouton sizes (Figures 1H, 3D, and 3E, inset). To determine the consequences for plasticity, we measured the change in weights after bursts of positively or negatively correlated input (see the Supplemental Experimental Procedures) (Figure 3F). Input bursts induced the most weight changes in our deprived model (Figures 3G and 3H). We reasoned that this increased potential for bidirectional plasticity may facilitate reorganization. Therefore, we compared the time taken for control (Figure 3I) and deprived (Figure 3K) networks to learn new input patterns (Figures 3I–3L). Simulations found deprived networks reached a saturated state of learning in response to novel input patterns faster than control networks (Figure 3M). Thus, our simulation suggests that reducing the range of correlated network activity may account for the reduced range of bouton sizes *in vivo* (likely leading to changes in both L2/3 input and output) and promote conditions of heightened bidirectional plasticity.

Enhanced Bidirectional Plasticity

Our network simulation predicted greater potential for bidirectional plasticity after size-dependent dynamics. The potential for synapses in deprived cortex to undergo enhanced synaptic strengthening and weakening may benefit reorganization. We used electrophysiology to examine these predictions. We made acute slices from deprived visual cortex at a time when imaging found the greatest reduction in bouton variance (16 days), and then we made whole-cell recordings from L2/3 pyramidal neurons (Figure 4A). To test the potential for neurons in L2/3 to undergo presynaptic plasticity, we first measured the paired pulse ratio (PPR) as an estimate of presynaptic release probability (Pr) (Regehr, 2012). For each neuron, we characterized the average PPR at a range of stimulation frequencies (1–10 Hz), inducing both paired pulse facilitation (PPF) and depression (PPD) (Figures 4B and 4C). The average PPR value was similar in control and deprived cortices (Figures 4B–4D, inset). However, the range of PPRs was reduced after deprivation so that the absolute difference of each PPR from the mean was less in deprived than control cortex (Figure 4D).

We next examined the degree of presynaptic plasticity expressed in deprived cortex by inducing a presynaptic form of either long-term potentiation (LTP) or depression (LTD), known to modify the PPR in cortical tissue (see the Supplemental Experimental Procedures) (Boudkkazi et al., 2007). Both LTP and LTD were greater in deprived than control cortex (Figures 4E and 4F). The induction of presynaptic LTP/LTD modified the PPR in control cortex, and these changes occurred in line with predicted shifts in Pr (Boudkkazi et al., 2007) (Figure 4G). After LTP induction, we observed a modest increase in PPD consistent with an increase in Pr, while LTD induction led to a small increase in PPF consistent with a decrease in Pr (Figure 4G). The changes in PPR after either LTP or LTD induction in deprived cortex were greater than those in control cortex (Figures 4H and 4I). Thus, our electrophysiology experiments support our network simulation suggesting size-dependent bouton dynamics are accompanied by greater bidirectional plasticity in deprived cortex.

DISCUSSION

We find persistent axonal boutons undergo size-dependent dynamics that reduce the range of bouton sizes in deprived

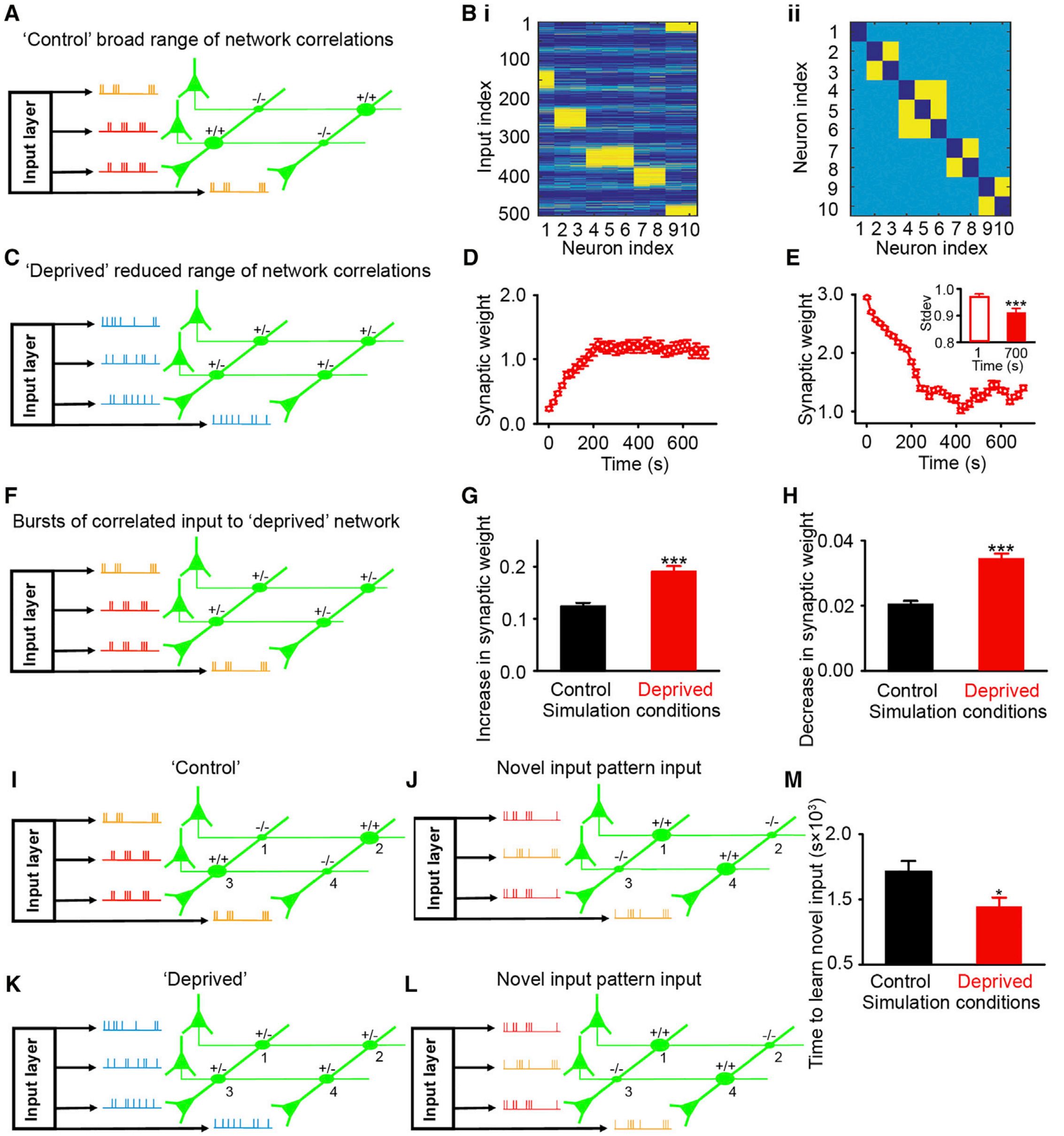

Figure 3. Network Simulation of Strength-Dependent Dynamics

(A) Control simulation. Addition and subtraction symbols show weight strengths between neurons.

(B) (i) Feedforward input weights into the neurons in the recurrent control network. Neurons 2 and 3 receive similar inputs, as do neurons 4–6, neurons 7 and 8, and neurons 9 and 10. Strong weights are yellow and weak weights are blue. (ii) Connection strengths from the presynaptic (x axis) to the postsynaptic neurons (y axis) within the control network. Neurons with correlated inputs are (yellow) bidirectionally connected, other neurons are (light blue) weakly connected, but not (dark blue) self-connected.

(C) Deprived simulation with reduced range of correlated input.

(D and E) The strength of weak weights (D) increases and the strength of strong weights (E) decreases (average of every 20th time point). (Inset) SD of all weights measured at time points 1 and 700 is shown.

(F) Schematic of plasticity simulation. Deprived model is presented with bursts of correlated input and the change in weights measured.

(G and H) Deprived networks show greater plasticity when given bursts of positively (G) or negatively (H) correlated input.

(I and J) Control network where weights (1–4) are established with highly correlated input (I) and then tested using novel input (J, i.e., a different set of neurons receives correlated inputs); weights 1 and 4 strengthen and weights 2 and 3 weaken.

(K and L) Schematic of deprived network (K) and testing with novel input patterns (L). In the cartoon, synaptic weights 1 and 4 strengthen and synaptic weights 2 and 3 weaken after new input.

(M) Deprived model learns novel input faster than control. For statistical comparisons and n values, see Table S3. *$p < 0.05$ and ***$p < 0.001$. Error bars, mean and SEM.

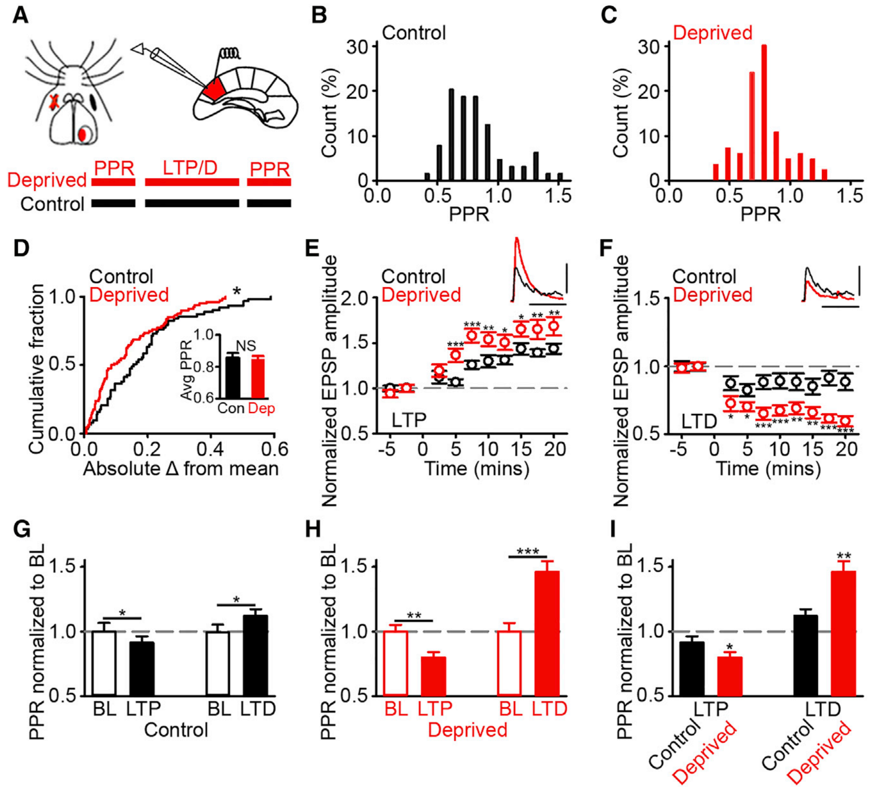

Figure 4. Enhanced Bidirectional Plasticity in Deprived Cortex

(A) Schematic of deprived mouse and slice electrophysiology in V1m L2/3. Timeline for the characterization of PPR, LTP/LTD induction, and PPR re-testing is shown.

(B and C) PPR in control (B) and deprived (C) cortices.

(D) Absolute difference of PPRs from population mean and (inset) average PPR in control and deprived cortices.

(E and F) LTP (E) and LTD (F) in deprived and control cortices. (Insets) Examples of excitatory postsynaptic potentials (EPSPs) from deprived cortex during baseline (E and F, black) and following either (red) LTP (E) or LTD (F) induction. Scale bars, 25 ms, 5 mV.

(G and H) Normalized change in PPR relative to baseline in control (G) and deprived (H) cortices after (left) LTP or (right) LTD.

(I) Change in PPR after induction of (left) LTP or (right) LTD in deprived and control cortex. Values are normalized to baseline. For statistical comparisons and n values, see Table S4. NS, not significant; $^{*}p < 0.05$, $^{**}p < 0.01$, and $^{***}p < 0.001$. Error bars, mean and SEM.

cortex. This process is accompanied by a reduced range of correlated network activity that when modeled predicts greater potential for bidirectional plasticity (i.e., synaptic strengthening and synaptic weakening). Electrophysiological experiments support these predictions, suggesting that size-dependent bouton dynamics may promote conditions beneficial for cortical reorganization.

Size-Dependent Bouton Dynamics

Previous work has focused mostly on bouton turnover, finding variable rates (De Paola et al., 2006; Majewska et al., 2006; Qiao et al., 2016). Persistent boutons are less studied but in aged animals exhibit large fluctuations in size, suggesting reduced synaptic tenacity with aging (Grillo et al., 2013). The activity underpinning such fluctuations is unclear. Our *in vivo* functional experiments combined with simulations suggest that a reduction in the range of correlated network activity shapes the distribution of persistent bouton sizes in deprived cortex, possibly because the reduced range of correlated activity no longer constrains or reinforces synapse strength. Size-dependent dynamics have been reported in a variety of preparations and may represent a general property of synapses (Minerbi et al., 2009; Loewenstein et al., 2011; Statman et al., 2014). Yet, few have investigated how sensory experience influences size-dependent dynamics. We find size-dependent dynamics to be most strongly expressed after deprivation, but we do not exclude the possibility that similar more subtle dynamics occur in naive animals (Loewenstein et al., 2011). Size/strength-dependent plasticity rules are widely used in theoretical models of network function (Kalantzis and Shouval, 2009; van Rossum et al., 2012), and they have been demonstrated *ex vivo* (Sáez and Friedlander, 2009). However, understanding of the *in vivo* biological mechanisms is limited.

Reduced Range of Network Correlations

We measured cortical activity with chronic calcium imaging, and we estimated the degree of correlated network activity with population coupling values (Okun et al., 2015). Population coupling reflects the strength of a neuron's relationship to the mean population activity, and it has been shown to correlate with the probability of local input in V1 (Okun et al., 2015). We found strongly coupled neurons became more weakly coupled after deprivation while weakly coupled neurons showed greater population coupling. Such changes in population coupling could be driven by factors including loss of patterned sensory activity, reduced inhibition (Barnes et al., 2015b), or changes in thalamic drive (Linden et al., 2009).

Measuring Persistent Boutons

Measuring presynaptic structures *in vivo* is challenging (Canty and De Paola, 2011). We adapted a published approach validated by *in vivo* imaging and ultra-structural analysis (Grillo et al., 2013). Our approach has limitations; for example, very small axonal varicosities may not harbor synapses. To exclude this possibility, we restricted our analysis to varicosities with intensities twice as great as the axon backbone, which always harbors synapses (Grillo et al., 2013). Our analysis is unable to distinguish between single and multi-synaptic boutons, however, only a small proportion of boutons make synaptic contacts with multiple spines (Knott et al., 2006). Finally, we studied

boutons from L2/3 and L5 pyramidal neurons as well as thalamocortical axons (Feng et al., 2000), but we do not know the identity of their post-synaptic targets. This is an important limitation with implications for circuit function (Lu et al., 2007; Jiang et al., 2015).

Consequences for the Network

The distribution of synaptic strengths is thought to encode sensory experience (Stepanyants and Escobar, 2011). Thus, the bouton changes we observe may result in the loss of stored sensory information. This process occurred over days and so information may be gradually degraded. Slow degradation may account for the ability to reinstate previous tuning properties when sensory deprivation is reversed, since a fraction of the prior weight distribution may remain (Rose et al., 2016). Although size-dependent bouton dynamics may result in the loss of information, this process may also carry functional benefit. This is because synapses with less extreme weights are thought to have greater potential to undergo both synaptic strengthening and synaptic weakening (van Rossum et al., 2012). In line with this, we found populations of synapses in deprived cortex underwent greater strengthening and weakening following LTP/LTD induction compared to controls. Therefore, the structural plasticity we describe may lead to a loss of stored sensory information but enhance the flexibility of synapses to adapt to novel non-deprived sensory input.

EXPERIMENTAL PROCEDURES

Further details and an outline of resources used in this work can be found in the Supplemental Experimental Procedures.

Animals

Experiments were conducted according to the UK Animals (Scientific Procedures) Act 1986. Male and female mice (post-natal day [P]60–P90) were sex and age matched within experimental groups, and they were housed with littermates on a 12-hr light-dark cycle. *Thy1*-GFP mice were used for structural imaging, and C57BL/6 mice were used for electrophysiology and were injected with AAV2/1-*ef1*α-GCaMP5 for functional imaging.

Statistics

Statistical analysis was performed in MATLAB or SigmaPlot. Comparisons were made using parametric or non-parametric statistics where appropriate. Correction for multiple testing used either Holm-Sidak or Dunn's method. Correlations were run using Pearson's correlation test.

SUPPLEMENTAL INFORMATION

Supplemental Information includes Supplemental Experimental Procedures and four tables and can be found with this article online at https://doi.org/10.1016/j.celrep.2017.12.065.

ACKNOWLEDGMENTS

Our work was supported by the Wellcome Trust 200790/Z/16/Z (to C.C.) and the Safra Foundation WMCTF30432 (to S.J.B.). The authors would like to thank Tara Keck and Georg Keller for functional imaging data; Andrew Lowe for code development; and Thomas Knöpfel, Vincenzo De Paola, Peter Haslehurst, and Gerald Finnerty for comments on the manuscript.

AUTHOR CONTRIBUTIONS

R.P.S., C.C., and S.J.B. designed and performed the experiments and wrote the paper.

DECLARATION OF INTERESTS

The authors declare no competing interests.

Received: October 9, 2017
Revised: December 4, 2017
Accepted: December 20, 2017
Published: January 16, 2018

REFERENCES

Akerboom, J., Chen, T.W., Wardill, T.J., Tian, L., Marvin, J.S., Mutlu, S., Calderón, N.C., Esposti, F., Borghuis, B.G., Sun, X.R., et al. (2012). Optimization of a GCaMP calcium indicator for neural activity imaging. J. Neurosci. *32*, 13819–13840.

Albieri, G., Barnes, S.J., de Celis Alonso, B., Cheetham, C.E., Edwards, C.E., Lowe, A.S., Karunaratne, H., Dear, J.P., Lee, K.C., and Finnerty, G.T. (2015). Rapid Bidirectional Reorganization of Cortical Microcircuits. Cereb. Cortex *25*, 3025–3035.

Barbour, B., Brunel, N., Hakim, V., and Nadal, J.-P. (2007). What can we learn from synaptic weight distributions? Trends Neurosci. *30*, 622–629.

Barnes, S.J., and Cheetham, C.E.J. (2014). A role for short-lived synapses in adult cortex? J. Neurosci. *34*, 7044–7046.

Barnes, S.J., Cheetham, C.E., Liu, Y., Bennett, S.H., Albieri, G., Jorstad, A.A., Knott, G.W., and Finnerty, G.T. (2015a). Delayed and Temporally Imprecise Neurotransmission in Reorganizing Cortical Microcircuits. J. Neurosci. *35*, 9024–9037.

Barnes, S.J., Sammons, R.P., Jacobsen, R.I., Mackie, J., Keller, G.B., and Keck, T. (2015b). Subnetwork-Specific Homeostatic Plasticity in Mouse Visual Cortex In Vivo. Neuron *86*, 1290–1303.

Barnes, S.J., Franzoni, E., Jacobsen, R.I., Erdelyi, F., Szabo, G., Clopath, C., Keller, G.B., and Keck, T. (2017). Deprivation-Induced Homeostatic Spine Scaling In Vivo Is Localized to Dendritic Branches that Have Undergone Recent Spine Loss. Neuron *96*, 871–882.e5.

Boudkkazi, S., Carlier, E., Ankri, N., Caillard, O., Giraud, P., Fronzaroli-Molinieres, L., and Debanne, D. (2007). Release-dependent variations in synaptic latency: a putative code for short- and long-term synaptic dynamics. Neuron *56*, 1048–1060.

Canty, A.J., and De Paola, V. (2011). Axonal reconstructions going live. Neuroinformatics *9*, 129–131.

Cheetham, C.E.J., Hammond, M.S.L., Edwards, C.E.J., and Finnerty, G.T. (2007). Sensory experience alters cortical connectivity and synaptic function site specifically. J. Neurosci. *27*, 3456–3465.

Cheetham, C.E., Barnes, S.J., Albieri, G., Knott, G.W., and Finnerty, G.T. (2014). Pansynaptic enlargement at adult cortical connections strengthened by experience. Cereb. Cortex *24*, 521–531.

Clopath, C., Büsing, L., Vasilaki, E., and Gerstner, W. (2010). Connectivity reflects coding: a model of voltage-based STDP with homeostasis. Nat. Neurosci. *13*, 344–352.

De Paola, V., Holtmaat, A., Knott, G., Song, S., Wilbrecht, L., Caroni, P., and Svoboda, K. (2006). Cell type-specific structural plasticity of axonal branches and boutons in the adult neocortex. Neuron *49*, 861–875.

Feng, G., Mellor, R.H., Bernstein, M., Keller-Peck, C., Nguyen, Q.T., Wallace, M., Nerbonne, J.M., Lichtman, J.W., and Sanes, J.R. (2000). Imaging neuronal subsets in transgenic mice expressing multiple spectral variants of GFP. Neuron *28*, 41–51.

Grillo, F.W., Song, S., Teles-Grilo Ruivo, L.M., Huang, L., Gao, G., Knott, G.W., Maco, B., Ferretti, V., Thompson, D., Little, G.E., and De Paola, V. (2013). Increased axonal bouton dynamics in the aging mouse cortex. Proc. Natl. Acad. Sci. USA *110*, E1514–E1523.

Jiang, X., Shen, S., Cadwell, C.R., Berens, P., Sinz, F., Ecker, A.S., Patel, S., and Tolias, A.S. (2015). Principles of connectivity among morphologically defined cell types in adult neocortex. Science *350*, aac9462.

Kalantzis, G., and Shouval, H.Z. (2009). Structural plasticity can produce metaplasticity. PLoS ONE *4*, e8062.

Keck, T., Mrsic-Flogel, T.D., Vaz Afonso, M., Eysel, U.T., Bonhoeffer, T., and Hübener, M. (2008). Massive restructuring of neuronal circuits during functional reorganization of adult visual cortex. Nat. Neurosci. *11*, 1162–1167.

Keck, T., Keller, G.B., Jacobsen, R.I., Eysel, U.T., Bonhoeffer, T., and Hübener, M. (2013). Synaptic scaling and homeostatic plasticity in the mouse visual cortex in vivo. Neuron *80*, 327–334.

Knott, G.W., Holtmaat, A., Wilbrecht, L., Welker, E., and Svoboda, K. (2006). Spine growth precedes synapse formation in the adult neocortex in vivo. Nat. Neurosci. *9*, 1117–1124.

Ko, H., Cossell, L., Baragli, C., Antolik, J., Clopath, C., Hofer, S.B., and Mrsic-Flogel, T.D. (2013). The emergence of functional microcircuits in visual cortex. Nature *496*, 96–100.

Linden, M.L., Heynen, A.J., Haslinger, R.H., and Bear, M.F. (2009). Thalamic activity that drives visual cortical plasticity. Nat. Neurosci. *12*, 390–392.

Loewenstein, Y., Kuras, A., and Rumpel, S. (2011). Multiplicative dynamics underlie the emergence of the log-normal distribution of spine sizes in the neocortex in vivo. J. Neurosci. *31*, 9481–9488.

Lu, J.T., Li, C.Y., Zhao, J.-P., Poo, M.M., and Zhang, X.H. (2007). Spike-timing-dependent plasticity of neocortical excitatory synapses on inhibitory interneurons depends on target cell type. J. Neurosci. *27*, 9711–9720.

Majewska, A.K., Newton, J.R., and Sur, M. (2006). Remodeling of synaptic structure in sensory cortical areas in vivo. J. Neurosci. *26*, 3021–3029.

Matsuzaki, M., Honkura, N., Ellis-Davies, G.C.R., and Kasai, H. (2004). Structural basis of long-term potentiation in single dendritic spines. Nature *429*, 761–766.

Meyer, D., Bonhoeffer, T., and Scheuss, V. (2014). Balance and stability of synaptic structures during synaptic plasticity. Neuron *82*, 430–443.

Minerbi, A., Kahana, R., Goldfeld, L., Kaufman, M., Marom, S., and Ziv, N.E. (2009). Long-term relationships between synaptic tenacity, synaptic remodeling, and network activity. PLoS Biol. *7*, e1000136.

Murthy, V.N., Schikorski, T., Stevens, C.F., and Zhu, Y. (2001). Inactivity produces increases in neurotransmitter release and synapse size. Neuron *32*, 673–682.

Noguchi, J., Nagaoka, A., Watanabe, S., Ellis-Davies, G.C.R., Kitamura, K., Kano, M., Matsuzaki, M., and Kasai, H. (2011). In vivo two-photon uncaging of glutamate revealing the structure-function relationships of dendritic spines in the neocortex of adult mice. J. Physiol. *589*, 2447–2457.

Okun, M., Steinmetz, N., Cossell, L., Iacaruso, M.F., Ko, H., Barthó, P., Moore, T., Hofer, S.B., Mrsic-Flogel, T.D., Carandini, M., and Harris, K.D. (2015). Diverse coupling of neurons to populations in sensory cortex. Nature *521*, 511–515.

Qiao, Q., Ma, L., Li, W., Tsai, J.-W., Yang, G., and Gan, W.-B. (2016). Long-term stability of axonal boutons in the mouse barrel cortex. Dev. Neurobiol. *76*, 252–261.

Regehr, W.G. (2012). Short-term presynaptic plasticity. Cold Spring Harb. Perspect. Biol. *4*, a005702.

Rose, T., Jaepel, J., Hübener, M., and Bonhoeffer, T. (2016). Cell-specific restoration of stimulus preference after monocular deprivation in the visual cortex. Science *352*, 1319–1322.

Sáez, I., and Friedlander, M.J. (2009). Plasticity between neuronal pairs in layer 4 of visual cortex varies with synapse state. J. Neurosci. *29*, 15286–15298.

Statman, A., Kaufman, M., Minerbi, A., Ziv, N.E., and Brenner, N. (2014). Synaptic size dynamics as an effectively stochastic process. PLoS Comput. Biol. *10*, e1003846.

Stepanyants, A., and Escobar, G. (2011). Statistical traces of long-term memories stored in strengths and patterns of synaptic connections. J. Neurosci. *31*, 7657–7669.

van Rossum, M.C.W., Shippi, M., and Barrett, A.B. (2012). Soft-bound synaptic plasticity increases storage capacity. PLoS Comput. Biol. *8*, e1002836.

Cell Reports, Volume *22*

Supplemental Information

Size-Dependent Axonal Bouton Dynamics following Visual Deprivation *In Vivo*

Rosanna P. Sammons, Claudia Clopath, and Samuel J. Barnes

SUPPLEMENTAL EXPERIMENTAL PROCEDURES

CONTACT FOR REAGENT AND RESOURCE SHARING

Further information and requests for resources and reagents should be directed to and will be fulfilled by the Lead Contact, Samuel Barnes (samuel.barnes@imperial.ac.uk).

EXPERIMENTAL MODEL AND SUBJECT DETAILS

Animals

Experiments were conducted according to the U.K. Animals (Scientific Procedures) Act 1986. Male and female mice (P60 – 90) were sex and age matched within experimental groups and housed with littermates on a 12 hr light-dark cycle. *Thy1*-GFP mice (Feng et al., 2000) were used for structural imaging, C57BL/6 mice for electrophysiology and C57BL/6 mice injected with AAV2/1-*ef1*α-GCaMP5 for functional imaging.

METHOD DETAILS

Animal Surgery

Cranial windows were surgically implanted over the right hemisphere of monocular visual cortex, as described previously (Barnes et al., 2015; Holtmaat et al., 2009). We made a craniotomy in ketamine (0.15 mg/g) /xylazine (0.015 mg/g) anesthetized mice and replaced the skull with a glass cover-slip that was attached to the bone with dental cement. Mice were allowed to recover for at least 21 days after surgery before imaging commenced. For monocular enucleation, we applied lidocaine to the area around the left eye prior to surgical removal of the eye. Monocular enucleation was conducted in adulthood (P60 – 90). Control 'sham-enucleated' mice were given time-matched anaesthesia. For structural imaging, animals were imaged while anaesthetised (under ketamine/xylazine) according to the experimental timeline **(Fig 1A)**. For functional imaging experiments, mice were injected with AAV2/1-*ef1*α-GCaMP5 before the glass cover slip was positioned and allowed to recover for 18 days prior to imaging experiments.

Structural imaging

Structural imaging was performed as described previously (Barnes et al., 2015). Briefly, we used a 2-P microscope with a MaiTai BB laser with a DeepSee prechirp unit (Newport/Spectra Physics) set to 909 nm and an Olympus 40× 0.8 NA water immersion objective. The average laser power was < 50 mW as measured at the back focal plane and was kept constant across imaging sessions. For image acquisition, we used Scanimage freeware (VidrioTech). Image acquisition parameters were: 64 × 64 µm, 512 × 512 pixels, 0.5 µm step in depth.

Functional imaging

Functional imaging was performed as described previously (Barnes et al., 2015; Keck et al., 2013; Keller et al., 2012) and data are taken from a previously published data set that was shared (Barnes et al., 2015). We performed different analyses on these data. In brief, functional calcium imaging was performed with a custom built 2-P microscope. The illumination source was a MaiTai eHP laser with a DeepSee prechirp unit (Newport/Spectra Physics) or a Chameleon Vision S (Coherent). The excitation wavelength was set to 910 nm. The scanhead was based on an 8 kHz resonant scanner (Cambridge Technology), used in bidirectional mode. This enabled frame rates of 40 Hz at 400 × 600 pixels. A high power objective Z-piezo stage (Physik Instrumente) was used in order to move the objective down in steps of approximately 20 µm between frames and return to the initial position after four frames. Data were acquired at four different depths, reducing the effective frame rate from 40 Hz to 10 Hz. Laser power was < 50 mW. We used a Nikon 16× 0.8 NA objective. Data were acquired with a 250 MHz digitizer (National Instruments) and pre-processed with a custom programmed FPGA (National Instruments). Head-fixed animals ran on a spherical treadmill while visual stimuli were presented on two screens arranged at 60 degrees relative to each other in front of the mouse. This arrangement is intended to simulate visual flow similar to that experienced when running between two walls. Visual stimuli were full field gratings, the motion of which was controlled by rotation of the spherical treadmill. Experiments consisted of alternating 3 minute blocks in which the mouse either received coupled visual

feedback or the screens were switched off. Each condition was repeated twice, followed by drifting gratings in eight directions (0-360 degrees by 45 degree steps) presented in a random order, with a spatial frequency of 0.04 cycles/degree and a temporal frequency of 2 Hz. The same visual stimulus paradigm was used at each time point before and after enucleation.

Network Simulation

The parameters for the model were kept fixed throughout all simulations and were taken from previously published work (Clopath et al., 2010; Ko et al., 2013). In order to train the network to the 'control' case, feedforward weights were initialized with receptive fields (weights taken from previous test simulations took values between 0 and 3 which were also the hard bounds). At the beginning of the simulation, recurrent excitatory connection weights were set to 1. The network settles in a configuration where neurons receiving correlated inputs develop strong inter-connectivity (Clopath et al., 2010; Ko et al., 2013). For example, in **Fig 3Bi,** neurons 2&3, neurons 4,5&6, and neurons 7&8, 9&10 receive correlated inputs indicated by the fact that they have strong feedforward weights to the same inputs, strong bidirectional lateral connectivity within their group and weak connections outside of their cluster. Note that neurons select their input preference randomly and that neurons were relabelled so that neighbouring neuron numbers are correlated. The model does not take into account physical space. In order to simulate the deprivation paradigm we reduced the range of correlated inputs to the network by replacing highly positively and highly negatively correlated inputs with uncorrelated inputs of the same strength so that neurons in the deprived simulation received the same statistics of input current as well as exhibited the same statistics of spiking as neurons in the control simulation. In particular, we kept the same feedforward weights and the same Poisson firing rate for the inputs as the control condition but every 100 ms, we randomly reallocated the inputs to half of the neurons.

For simulations of network plasticity, we injected input patterns changing every 100 ms and recorded the total positive weight change and the total negative weight change over 1000 s. The simulations were run 50 times. In order to simulate the time it takes for the network to learn a new pattern we removed the inputs to the network (either the control or the deprived network) and replaced them with other correlated inputs of the same strength and correlation level as the control case (e.g. instead of in **Fig 3Bi** neurons 2&3, 4,5&6, and 7&8, 9&10 being correlated, we chose another random assignment such as 2&4, 6&1, 3&10, 5,7&8). We then measured the time it took for the network to learn the new pattern. In particular, we measured how long it took for the lateral weights to become strong within their correlated groups (more than two thirds of their maximal size) and weak across groups (less than a third of the maximal size). The simulations were run 50 times. In a separate simulation we lowered activity in the deprived case rather than reducing the range of correlated network activity. In this alternate model, we found that both weak (0.67 ± 0.10, Normalized Δ in weights, $p = 0.002$, paired t-test) and strong (0.67 ± 0.03, Normalized Δ in weights, $p < 0.001$, paired t-test) synaptic weights decreased in strength.

Electrophysiology

Electrophysiological recordings were conducted as previously described (Barnes et al., 2015). Deeply anaesthetized mice were transcardially perfused with 10 ml of ice-cold (4 ºC) dissection artificial cerebral spinal fluid (ASCF; in mM, 108 choline-Cl, 3 KCl, 26 $NaHCO_3$, 1.25 $NaHPO_4$, 25 D-glucose, 3 Na pyruvate, 2 $CaCl_2$ and 1 $MgCl_2$ saturated with 95% O_2 / 5% CO_2). Coronal brain slices 300 µm thick were cut (Vibratome 3000, Leica) from visual cortex. Slices were incubated for at least 60 minutes in a holding chamber and then recordings were made at room temperature (24 ºC) in a recording ASCF (in mM, 120 NaCl, 3 KCl, 23 $NaHCO_3$, 1.25 $NaHPO_4$, 10 D-glucose, 2 $CaCl_2$ and 1 $MgCl_2$ saturated with 95% O_2 / 5% CO_2). Recordings were targeted to V1m based on stereotaxic coordinates. We recorded from L2/3 on a custom-built microscope under infrared differential interference contrast microscopy. L2/3 pyramidal neurons were identified based on spiking properties and pyramidal shaped soma and recorded in current clamp mode (Multiclamp 700B, Molecular Devices), using Ephus acquisition software (VidrioTech). We did not correct for the liquid junction potential. Patch pipettes (4 – 7 MΩ) contained the following in mM: 130 $KMeSO_3$, 8 NaCl, 2 KH_2PO_4, 2 D-glucose and 10 HEPES. The paired pulse ratio (PPR) values of L2/3 pyramidal neurons were assessed in whole-cell mode using stimulation of horizontal L2/3 inputs with a concentric bipolar stimulating electrode. Extracellular

stimulation at 1 – 10 Hz consisted of 1 ms current pulses with a stimulation intensity set to 50 % of the intensity that evoked the maximum EPSP amplitude. PPR values at four frequencies (1, 2, 5 and 10 Hz) were taken as the average of 20 trials per neuron. LTP and LTD were induced using an adapted method (Boudkkazi et al., 2007). LTD was induced by a low frequency stimulation protocol in which the stimulation was applied at 3 Hz for 3 – 5 minutes whilst the post-synaptic neuron was held at a membrane potential of -40 mV. LTP was induced by stimulating at 1 Hz during 2-3 minutes whilst the postsynaptic neuron was held at -10 mV. All Electrophysiology data was analysed by custom written software in Matlab.

QUANTIFICATION AND STATISTICAL ANALYSIS

Structural imaging Analysis

In vivo 2P images were processed in ImageJ (US National Institutes of Health, Bethesda, MD) and custom written software in Matlab (Mathworks). Fluorescence intensity profiles of axonal stretches including boutons were measured at multiple depths in ImageJ and the three maximum intensity profiles were averaged for each bouton **(Fig 1B)**. The average fluorescence intensity profile was then processed by semi-automated custom software in Matlab. The baseline axonal backbone was binned according to fluorescence intensity and fitted with a linear function and then subtracted to leave the average bouton intensity profile. Individual bouton profiles were then fitted with Gaussian curves and the area under the curve was normalised to the intensity of the axonal backbone so that raw bouton size values in Figure 1 are given in axonal backbone units (unless otherwise stated). All boutons were checked against the original images by two experimenters who were blind to the experimental condition to ensure that boutons were reliably identified as, persistent, disappearing or new. To be included in the analysis boutons had to be at least twice as bright as the background in at least one session, this criteria is based on previous correlative EM and *in vivo* imaging findings which adopted similar normalisation techniques (Grillo et al., 2013). Boutons were defined as persistent if they were present in all imaging timepoints. We sampled similar numbers of boutons per mouse in deprived (43 ± 7, n = 8 mice) and control (42 ± 7, n = 8 mice, p = 0.911, t-test) animals. Furthermore, the average length of axon sampled per mouse was also similar between deprived (22 ± 2, n = 8 mice) and control (24 ± 1, n = 8 mice, p = 0.162, t-test) animals. The initial (0 d) size of the bouton in axonal backbone units was used to define boutons as either small, mid-range or large. Boutons were considered small if their initial size was in the lowest 30th percentile of bouton sizes; large boutons were defined as those whose initial size was in the top 70th to 100th percentile. Those boutons whose size lay between the 30th and 70th percentiles were classified as mid-range. The average bouton size was similar prior to deprivation in control and deprived cortex **(Fig 1Si)**. The bouton turnover ratio (TOR) was calculated using previously published criteria where the TOR between two imaging sessions was defined as (nNew + nDisp)/2N, where nNew and nDisp are the number of new and disappearing boutons respectively and N is the total number of boutons in a session (Grillo et al., 2013).

Functional imaging and analysis

Processing of raw functional imaging data was as described previously (Barnes et al., 2015; Keck et al., 2013) For functional data analysis cells were selected based on mean and maximum projections of the data by hand (the nucleus was excluded from the selection) and high resolution depth stacks were used to ensure that cells did not have a filled nucleus. Fluorescence traces were calculated as the average fluorescence of pixels lying within the cell in each frame. To remove slow signal changes in raw fluorescence traces, the 8th-percentile value of the fluorescence distribution in a ± 15 second window was subtracted from the raw fluorescence signal (Dombeck et al., 2007). Calcium signals (%$\Delta F/F_0$) were calculated by dividing the raw fluorescence signal by the median of each cell's fluorescence distribution. Cellular activity was calculated using the integrated fluorescence as describe previously (Barnes et al., 2015) with a 15 % $\Delta F/F_0$ threshold. Activity was then normalized to the duration of our imaging paradigm to give values in %$\Delta F/F_0$/second. For each imaged region we calculated the mean and the variance of the activity values. Measurements of population coupling were estimated using the 'Pearson Coupling' approach (Okun et al., 2015). We first generated an average population rate trace for each neuron by taking the average fluorescence signal at each time bin for all neurons in a region (excluding the neuron of interest). We then ran Pearson pairwise correlations between the average population rate trace and each neurons activity trace in an imaged region. For each imaged region we calculated the mean and the variance of the population coupling scores. In order to normalize values and pool data across experiments we

took the difference (or absolute difference) between each neurons' population coupling score and the average population coupling score in that imaged region.

Statistics

Statistical analyses were performed either in Matlab or SigmaPlot. Data were tested for equal variance and normality (Shapiro-Wilk test) and then comparisons were made using parametric or non-parametric tests, as appropriate (t-test, paired t-test, Chi-square test, Mann-Whitney Rank Sum test, One-Way ANOVA with Holm-Sidak post-hoc test, Repeated measures ANOVA with Holm-Sidak post-hoc test, or a Two-Way ANOVA with Holm-Sidak post-hoc test). For normalized axonal bouton size, data were log_{10} transformed (Loewenstein et al., 2011) as noted in the text. Statistical tests were two-sided. Correlation coefficients were calculated with Pearson's correlation coefficient. A power analysis was performed to ensure we used a sufficient sample size. Specific statistical tests used for all figures along with the number of samples and details of center and dispersion measures can be found in supplemental tables S1-S4.

DATA AND SOFTWARE AVAILABILITY

Requests for data and software should be directed to the Lead Contact, Samuel Barnes (samuel.barnes@imperial.ac.uk) and will be made available upon reasonable request.

Code availability

The code used for image registration and data acquisition of the functional data is available at: http://sourceforge.net/projects/iris-scanning/

RESOURCES TABLE

REAGENT or RESOURCE	SOURCE	IDENTIFIER
Bacterial and Virus Strains		
AAV2/1-*ef1*α-GCaMP5	FMI Vector Core	N/A
Chemicals, Peptides, and Recombinant Proteins		
Isoflurane (Attane)	Provet	CAS 26675-46-7
Dental Cement (Paladur)	Heraeus Kulzer	CAS 9066-86-8
Ketamine	Pfizer	CAS 1867-66-9
Xylazine	Rompun	CAS 7361-61-7
Emla Cream 5 %	AstraZeneca	CAS 137-58-6<br>CAS 721-50-6
D Glucose	Sigma-Aldrich	CAS 50-99-7
NaCl,	Tocris	CAS 7647-14-5
KCl	Tocris	CAS 7447-40-7
$NaHCO_3$	Tocris	CAS 144-55-8
NaH_2PO_4	Tocris	CAS 7558-80-7
$CaCl_2$	Sigma-Aldrich	CAS 10043-52-4
$MgSO_4$	Sigma-Aldrich	CAS 7487-88-9
$KMeSO_3$	Sigma-Aldrich	CAS 562-54-9
KH_2PO_4	Sigma-Aldrich	CAS 7778-77-0
Na pyruvate	Sigma-Aldrich	CAS 113-24-6
Choline-Cl	Sigma-Aldrich	CAS 67-48-1
HEPES	Sigma-Aldrich	CAS 7365-45-9
Experimental Models: Organisms/Strains		
Mouse: Thy-1 GFP-M line	JAX	RRID:IMSR_JAX:007788
Mouse: C57BL6/J	Charles River Laboratories	N/A
Software and Algorithms		
Matlab	The MathWorks, Inc.	RRID: SCR_001622

LabView	National Instruments	RRID: SCR_014325
Sigmaplot13	Systat Software, Inc.	N/A
Scanimage	Vidrio technolgies	RRID: SCR_014307
Ephus	Vidrio technolgies	N/A
ImageJ	NIH	RRID: SCR_003070

REFERENCES

Barnes, S.J., Sammons, R.P., Jacobsen, R.I., Mackie, J., Keller, G.B., and Keck, T. (2015). Subnetwork-Specific Homeostatic Plasticity in Mouse Visual Cortex In Vivo. Neuron *86*, 1290–1303.

Boudkkazi, S., Carlier, E., Ankri, N., Caillard, O., Giraud, P., Fronzaroli-Molinieres, L., and Debanne, D. (2007). Release-dependent variations in synaptic latency: a putative code for short- and long-term synaptic dynamics. Neuron *56*, 1048–1060.

Clopath, C., Büsing, L., Vasilaki, E., and Gerstner, W. (2010). Connectivity reflects coding: a model of voltage-based STDP with homeostasis. Nat. Neurosci. *13*, 344–352.

Dombeck, D.A., Khabbaz, A.N., Collman, F., Adelman, T.L., and Tank, D.W. (2007). Imaging large-scale neural activity with cellular resolution in awake, mobile mice. Neuron *56*, 43–57.

Feng, G., Mellor, R.H., Bernstein, M., Keller-Peck, C., Nguyen, Q.T., Wallace, M., Nerbonne, J.M., Lichtman, J.W., and Sanes, J.R. (2000). Imaging neuronal subsets in transgenic mice expressing multiple spectral variants of GFP. Neuron *28*, 41–51.

Grillo, F.W., Song, S., Teles-Grilo Ruivo, L.M., Huang, L., Gao, G., Knott, G.W., Maco, B., Ferretti, V., Thompson, D., Little, G.E., et al. (2013). Increased axonal bouton dynamics in the aging mouse cortex. Proc. Natl. Acad. Sci. U. S. A. *110*, E1514-1523.

Holtmaat, A., Bonhoeffer, T., Chow, D.K., Chuckowree, J., De Paola, V., Hofer, S.B., Hübener, M., Keck, T., Knott, G., Lee, W.-C.A., et al. (2009). Long-term, high-resolution imaging in the mouse neocortex through a chronic cranial window. Nat. Protoc. *4*, 1128–1144.

Keck, T., Keller, G.B., Jacobsen, R.I., Eysel, U.T., Bonhoeffer, T., and Hübener, M. (2013). Synaptic scaling and homeostatic plasticity in the mouse visual cortex in vivo. Neuron *80*, 327–334.

Keller, G.B., Bonhoeffer, T., and Hübener, M. (2012). Sensorimotor mismatch signals in primary visual cortex of the behaving mouse. Neuron *74*, 809–815.

Ko, H., Cossell, L., Baragli, C., Antolik, J., Clopath, C., Hofer, S.B., and Mrsic-Flogel, T.D. (2013). The emergence of functional microcircuits in visual cortex. Nature *496*, 96–100.

Loewenstein, Y., Kuras, A., and Rumpel, S. (2011). Multiplicative dynamics underlie the emergence of the log-normal distribution of spine sizes in the neocortex in vivo. J. Neurosci. Off. J. Soc. Neurosci. *31*, 9481–9488.

Okun, M., Steinmetz, N., Cossell, L., Iacaruso, M.F., Ko, H., Barthó, P., Moore, T., Hofer, S.B., Mrsic-Flogel, T.D., Carandini, M., et al. (2015). Diverse coupling of neurons to populations in sensory cortex. Nature *521*, 511–515.

Panel	Comparison		Test	Control	Deprived	p value	n value
**1C**	Axon software vs Summed volume		Linear Regression	R^2 = 0.93		p < 0.001	n = 19 Boutons
**1C Inset**	Δ axonal backbone units over time 0-0.5 vs 0-6.0 hrs		t-test	0-0.5 hrs 2.2 ± 0.3 vs 0-6.0 hrs 11.1 ± 2.0		p < 0.001	n = 19 Boutons
**1D**	Bouton Turnover Ratio Control vs Deprived	3 d	Chi-square test	0.08	0.12	p = 0.017	Control = 55 axons Deprived = 62 axons
		6 d		0.05	0.06	p = 0.439	
		9 d		0.03	0.04	p = 0.190	
		16 d		0.06	0.04	p = 0.272	
**1D Inset**	Disappearing Boutons (Control vs Deprived)		Chi-square test	11 %	17 %	p = 0.010	Control = 55 axons Deprived = 62 axons
	New Boutons (Control vs Deprived)		Chi-square test	5 %	7 %	p = 0.235	
**1E**	Bouton size distribution		Log normal fit		R^2 = 0.90	p < 0.001	Deprived =345 Boutons
**1F**	Log transformed bouton size distribution		Gaussian fit		R^2 = 0.96	p < 0.001	
**1G**	Average of persistent bouton sizes (Log_{10}) per region (vs 0 d)	0 d	One-Way Repeated measures ANOVA	1.46 ± 0.07	1.52 ± 0.05	Deprived p = 0.074 Control p = 0.908	Control = 11 regions Deprived = 10 regions
		3 d		1.47 ± 0.08	1.47 ± 0.05		
		6 d		1.46 ± 0.08	1.41 ± 0.04		
		9 d		1.49 ± 0.09	1.50 ± 0.05		
		16 d		1.48 ± 0.07	1.50 ± 0.04		
**1H**	Variance of persistent bouton sizes (Log_{10}) per region (vs 0 d)	0 d	One-Way Repeated measures ANOVA	0.099 ± 0.014	0.110 ± 0.007	Deprived 0 vs 3 d p = 0.019 0 vs 6 d p = 0.031 0 vs 9 d p = 0.007 0 vs 16 d p = 0.004 Control p = 0.908	
		3 d		0.110 ± 0.017	0.086 ± 0.008		
		6 d		0.111 ± 0.018	0.085 ± 0.004		
		9 d		0.116 ± 0.017	0.078 ± 0.006		
		16 d		0.110 ± 0.014	0.076 ± 0.004		

Statistical Comparisons for Figure 1 I – O				Group (Center and dispersion or fit)		Result	
**Panel**	**Comparison**		**Test**	**Control**	**Deprived**	**p value**	**n value**
**1I**	Log transformed bouton size distribution		Gaussian fit		R^2 = 0.96	p < 0.001	Control = 343 Boutons<br>Deprived = 345 Boutons
**1K**				R^2 = 0.93		p < 0.001	
**1J**	Difference in bouton size distributions (%age count)		Descriptive		< 25th = -8 %<br>25th to 75th = +9 %<br>> 75th = -12 %	N/A	
**1L**				< 25th = +1 %<br>25th to 75th = -8 %<br>> 75th = +1 %			
**1L inset**	Absolute Δ of each bouton from population mean of Log_{10} bouton distribution		One-Way ANOVA	Baseline (0 d)<br>0.27 ± 0.01<br>vs<br>Control (16 d)<br>0.29 ± 0.02	Baseline (0 d)<br>0.27 ± 0.01<br>vs<br>Deprived (16 d)<br>0.21 ± 0.01	Deprived<br>p = 0.007<br><br>Control<br>p = 0.337	
**1M**	Change in bouton size normalized to 0 d<br><br>For boutons of different sizes		Two-Way ANOVA	Small<br>1.07 ± 0.04	Small<br>1.26 ± 0.06	p < 0.001	
				Medium<br>1.02 ± 0.02	Medium<br>1.00 ± 0.03	p = 0.688	
				Large<br>0.95 ± 0.02	Large<br>0.86 ± 0.02	p = 0.045	
**1N**	Average size of large boutons over time<br>(vs 0 d)<br><br>(Normalized axonal backbone units)	0 d	One-Way Repeated measures ANOVA	77 ± 5	69 ± 3	Deprived<br>0 vs 3 d<br>p < 0.001<br><br>0 vs 6 d<br>p < 0.001<br><br>0 vs 9 d<br>p < 0.001<br><br>0 vs 16 d<br>p < 0.001<br><br>Control<br>p = 0.161	
		3 d		68 ± 6	53 ± 4		
		6 d		65 ± 7	42 ± 3		
		9 d		72 ± 6	48 ± 3		
		16 d		65 ± 5	43 ± 3		
**1O**	Average size of small boutons over time<br>(vs 0 d)<br><br>(Normalized axonal backbone units)	0 d	One-Way Repeated measures ANOVA	16 ± 1	17 ± 1	Deprived<br>0 vs 3 d<br>p = 0.002<br><br>0 vs 6 d<br>p = 0.007<br><br>0 vs 9 d<br>p < 0.001<br><br>0 vs 16 d<br>p < 0.001<br><br>Control<br>p = 0.154	
		3 d		18 ± 2	25 ± 2		
		6 d		18 ± 1	24 ± 2		
		9 d		17 ± 1	32 ± 3		
		16 d		20 ± 1	33 ± 3		

Statistical Comparisons for Figure 1 P – S			Group (Center and dispersion or fit)		Result	
**Panel**	**Comparison**	**Test**	**Control**	**Deprived**	**p value**	**n value**
**1P**	Comparison of sizes at 16 d to medium size at 16 d<br>(sizes were initially binned based on baseline values 0 d)	One-Way ANOVA	Small (16 d)<br>20 ± 2<br>vs<br>Medium (16 d)<br>37 ± 3	Small (16 d)<br>31 ± 2<br>vs<br>Medium (16 d)<br>35 ± 4	Deprived<br>p = 0.778<br><br>Control<br>p = 0.003	Control = 343 Boutons<br><br>Deprived = 345 Boutons
			Large (16 d)<br>65 ± 5<br>vs<br>Medium (16 d)<br>37 ± 3	Large (16 d)<br>43 ± 3<br>vs<br>Medium (16 d)<br>35 ± 4	Deprived<br>p = 0.106<br><br>Control<br>p < 0.001	
**1Q**	Bouton size vs rate of change between imaging sessions	Pearson Correlation		r = -0.51	p < 0.001	
			r = -0.23		p < 0.001	
**1R**	Rate of change<br>0 – 3 d vs 9 – 16 d<br>(absolute rate of change in bouton size Log$_{10}$/day)	MWRST		0 – 3 d<br>0.07, 0.03 – 0.10<br>vs<br>9 – 16 d<br>0.04, 0.01 – 0.07	Deprived<br>p < 0.001	
**1Si**	Bouton size (0 d)<br><br>(Normalized axonal backbone units)	t-test	43 ± 3	42 ± 2	p = 0.725	
**1Sii**	Bouton variance (0 d)<br><br>(Normalized axonal backbone units)		22 ± 3	22 ± 2	p = 0.893	Control = 11 regions<br><br>Deprived = 10 regions
Control = 343 boutons from 55 axons measured in 8 mice (42 ± 7 boutons/mouse)<br>Deprived = 345 boutons from 62 axons measured in 8 mice (43 ± 7 boutons/mouse)						

Table S1. Statistical comparisons for Figure 1, Related to Figure 1.

Statistical Comparisons<br>for Figure 2 B – K			Group<br>(Center and dispersion or fit)		Result	
**Panel**	**Comparison**	**Test**	**Control**	**Deprived**	**p value**	**n value**
**2B**	Average activity<br>per region<br>compared to baseline<br>(Normalized)	One-Way<br>Repeated<br>Measures<br>ANOVA	0 d<br>1.00 ± 0.02<br>vs<br>3 d<br>0.99 ± 0.02	0 d<br>1.00 ± 0.02<br>vs<br>3 d<br>0.94 ± 0.02	p = 0.144	
**2B**	Average activity<br>per region<br>Control vs Deprived<br>(Normalized)	t-test	3 d<br>0.99 ± 0.02	3 d<br>0.94 ± 0.02	p = 0.141	
**2C**	Variance of activity<br>per region<br>compared to baseline<br>(Normalized)	One-Way<br>Repeated<br>Measures<br>ANOVA	0d<br>1.00 ± 0.04<br>vs<br>3 d<br>1.15 ± 0.04	0 d<br>1.00 ± 0.06<br>vs<br>3 d<br>1.05 ± 0.05	p = 0.096	Deprived<br>n = 32 regions<br><br>Control<br>n = 32 regions
**2C**	Variance of activity<br>per region<br>Control vs Deprived<br>(Normalized)	t-test	3 d<br>1.15 ± 0.04	3 d<br>1.05 ± 0.05	p = 0.144	
**2E**	Mean population<br>coupling scores<br>per region<br>compared to baseline<br>(Normalized)	One-Way<br>Repeated<br>Measures<br>ANOVA	0 d<br>1.00 ± 0.03<br>vs<br>3 d<br>1.03 ± 0.03	0 d<br>1.00 ± 0.03<br>vs<br>3 d<br>1.05 ± 0.03	p = 0.469	
**2E**	Mean population<br>coupling scores<br>per region<br>Control vs Deprived<br>(Normalized)	t-test	3 d<br>1.03 ± 0.03	3 d<br>1.05 ± 0.03	p = 0.660	
**2F**	Variance of<br>population coupling<br>scores per region<br>compared to baseline<br>(Normalized)	One-Way<br>Repeated<br>measures<br>ANOVA	0 d<br>1.00 ± 0.07<br>vs<br>3 d<br>0.92 ± 0.06	0 d<br>1.00 ± 0.07<br>vs<br>3 d<br>0.76 ± 0.04	Deprived<br>p = 0.017<br><br>Control<br>p = 0.534	
**2F**	Variance of<br>population coupling<br>scores per region<br>Control vs Deprived<br>(Normalized)	t-test	3 d<br>0.92 ± 0.06	3 d<br>0.76 ± 0.04	p = 0.033	
**2I**	Difference in<br>normalized<br>population coupling<br>distributions	Descriptive		< 25th = -7 %<br>25th to 75th = +11 %<br>> 75th = -6 %	N/A	
**2J**	Absolute difference<br>from population<br>coupling mean<br>(0 vs 3 d)	MWRST		0 d<br>0.15, 0.08 – 0.22<br>vs<br>3 d<br>0.12, 0.06 – 0.19	p < 0.001	
**2K**			0 d<br>0.13, 0.07 –<br>0.19<br>vs<br>3 d<br>0.13, 0.07 –<br>0.18		p = 0.248	

Statistical Comparisons for Figure 2 L – M			Group (Center and dispersion or fit)		Result	
**Panel**	**Comparison**	**Test**	**Control**	**Deprived**	**p value**	**n value**
**2L**	Weakly coupled Neurons (-0.5 to -0.1) compared to baseline (0 vs 3 d)	Paired t-test		0 d<br>-0.184 ± 0.003<br>vs<br>3 d<br>-0.059 ± 0.007	$p < 0.001$	Deprived<br>n = 32 regions<br><br>Control<br>n = 32 regions
**2L**	Strongly coupled Neurons (+0.2 to +0.5) compared to baseline (0 vs 3 d)	Paired t-test		0 d<br>0.323 ± 0.006<br>vs<br>3 d<br>0.082 ± 0.019	$p < 0.001$	
**2M**	Weakly coupled Neurons Control vs Deprived (3 d)	t-test	3 d<br>-0.101 ± 0.007	3 d<br>-0.059 ± 0.007	$p < 0.001$	
**2M**	Strongly coupled Neurons Control vs Deprived (3 d)	t-test	3 d<br>0.184 ± 0.015	3 d<br>0.082 ± 0.019	$p < 0.001$	
Deprived = 1081 neurons from 32 regions in 4 animals<br>Control = 1155 neurons from 32 regions in 4 animals						

Table S2. Statistical comparisons for Figure 2, Related to Figure 2.

Statistical Comparisons for Figure 3 D – M			Group (Center and dispersion or fit)		Result	
**Panel**	**Comparison**	**Test**	**Control**	**Deprived**	**p value**	**n value**
**3D**	Δ strength of weak weights	Paired t-test		Timepoint 1<br>0.23 ± 0.01<br>vs<br>Timepoint 700<br>1.11 ± 0.04	$p < 0.001$	Synaptic weights = 100<br><br>Trials = 100<br><br>700 or 1000 timepoints
**3E**	Δ strength of strong weights	Paired t-test		Timepoint 1<br>2.97 ± 0.01<br>vs<br>Timepoint 700<br>1.40 ± 0.02	$p < 0.001$	
**3E inset**	Standard deviation of all synaptic weights across stimulation<br><br>Timepoint 1<br>vs<br>Timepoint 700	Paired t-test		Timepoint 1<br>0.97 ± 0.01<br>vs<br>Timepoint 700<br>0.91 ± 0.02	$p < 0.001$	
**3G**	Increase in synaptic weights following positively correlated bursts	t-test	0.125 ± 0.006	0.192 ± 0.009	$p < 0.001$	Synaptic weights = 100<br><br>Input trials = 50
**3H**	Decrease in synaptic weights following negatively correlated bursts	t-test	0.021 ± 0.001	0.035 ± 0.002	$p < 0.001$	
**3M**	Time to learn novel patterns (seconds x 10^3):	t-test	1.72 ± 0.01	1.45 ± 0.01	$p = 0.012$	Synaptic weights = 100<br><br>Patterns = 50
Network simulation = 100 synaptic weights, run for 100 trials over 700 or 1000 timepoints.						

Table S3. Statistical comparisons for Figure 3, Related to Figure 3.

Statistical Comparisons for Figure 4 D – I				Group (Center and dispersion or fit)		Result	
**Panel**	**Comparison**		**Test**	**Control**	**Deprived**	**p value**	**n value**
**4D inset**	Average PPR value		t-test	0.86 ± 0.03	0.85 ± 0.02	p = 0.801	Control = 15 neurons<br><br>Deprived = 20 neurons
**4D**	Range of PPR values (absolute Δ from population mean)		MWRST	0.16, 0.07 – 0.23	0.09, 0.05 – 0.21	p = 0.028	
**4E**	LTP Control vs Deprived (Normalized values)	-5 mins	Two-Way ANOVA	1.00 ± 0.03	0.94 ± 0.04	p = 0.507	Control = 21 neurons<br><br>Deprived = 24 neurons
		-2.5 mins		1.00 ± 0.04	1.00 ± 0.04	p = 0.968	
		+2.5 mins		1.12 ± 0.07	1.19 ± 0.07	p = 0.426	
		+5 mins		1.07 ± 0.03	1.37 ± 0.07	p < 0.001	
		+7.5 mins		1.26 ± 0.05	1.58 ± 0.07	p < 0.001	
		+10 mins		1.30 ± 0.07	1.54 ± 0.08	p = 0.005	
		+12.5 mins		1.31 ± 0.05	1.50 ± 0.09	p = 0.029	
		+15 mins		1.44 ± 0.06	1.65 ± 0.08	p = 0.014	
		+17.5 mins		1.39 ± 0.04	1.65 ± 0.10	p = 0.006	
		+20 mins		1.44 ± 0.06	1.69 ± 0.10	p = 0.007	
**4F**	LTD Control vs Deprived (Normalized values)	-5 mins	Two-Way ANOVA	1.00 ± 0.04	0.99 ± 0.03	*p* = 0.827	
		-2.5 mins		1.00 ± 0.03	1.00 ± 0.03	*p* = 0.944	
		+2.5 mins		0.88 ± 0.05	0.73 ± 0.06	*p* = 0.029	
		+5 mins		0.82 ± 0.05	0.70 ± 0.04	*p* = 0.049	
		+7.5 mins		0.88 ± 0.05	0.65 ± 0.04	*p* < 0.001	
		+10 mins		0.89 ± 0.06	0.67 ± 0.04	*p* < 0.001	
		+12.5 mins		0.89 ± 0.06	0.69 ± 0.04	*p* = 0.003	
		+15 mins		0.85 ± 0.06	0.66 ± 0.04	*p* = 0.003	
		+17.5 mins		0.91 ± 0.05	0.62 ± 0.03	*p* < 0.001	
		+20 mins		0.89 ± 0.06	0.60 ± 0.04	*p* < 0.001	
**4G**	Δ in PPR after LTP (normalized to baseline BL)		Paired t-test	BL 1.00 ± 0.07 vs Post LTP 0.91 ± 0.05		p = 0.032	
**4H**					BL 1.00 ± 0.05 vs Post LTP 0.80 ± 0.04	p = 0.003	
**4G**	Δ in PPR after LTD (normalized to baseline BL)		Paired t-test	BL 1.00 ± 0.06 vs Post LTD 1.12 ± 0.06		p = 0.025	
**4H**					BL 1.00 ± 0.07 vs Post LTD 1.46 ± 0.08	p < 0.001	
**4I**	Δ in PPR after LTP Control vs Deprived On Log10 transformed data		t-test	Post LTP 0.91 ± 0.05	Post LTP 0.80 ± 0.04	p = 0.046	
	Δ in PPR after LTD Control vs Deprived		t-test	Post LTD 1.12 ± 0.06	Post LTD 1.46 ± 0.08	p = 0.002	
Figure 4 B-D uses 15 control and 20 deprived neurons from 8 control and 8 deprived animals.<br>Figure 4 E-I uses 21 control and 24 deprived neurons from 7 control and 7 deprived animals.							

Table S4. Statistical comparisons for Figure 4, Related to Figure 4.
